# A novel role for the histone acetyltransferase Hat1 in the CENP-A/CID assembly pathway in *Drosophila melanogaster*

**DOI:** 10.1093/nar/gkv1235

**Published:** 2015-11-19

**Authors:** Mark Boltengagen, Anming Huang, Anastasiya Boltengagen, Lukas Trixl, Herbert Lindner, Leopold Kremser, Martin Offterdinger, Alexandra Lusser

**Affiliations:** 1 Division of Molecular Biology, Biocenter, Medical University of Innsbruck, 6020 Innsbruck, Austria; 2 Division of Clinical Biochemistry, Biocenter, Medical University of Innsbruck, 6020 Innsbruck, Austria; 3 Division of Neurobiochemistry, Biocenter, Medical University of Innsbruck, 6020 Innsbruck, Austria

## Abstract

The incorporation of CENP-A into centromeric chromatin is an essential prerequisite for kinetochore formation. Yet, the molecular mechanisms governing this process are surprisingly divergent in different organisms. While CENP-A loading mechanisms have been studied in some detail in mammals, there are still large gaps to our understanding of CENP-A/Cid loading pathways in *Drosophila*. Here, we report on the characterization and delineation of at least three different CENP-A preloading complexes in *Drosophila*. Two complexes contain the CENP-A chaperones CAL1, FACT and/or Caf1/Rbap48. Notably, we identified a novel complex consisting of the histone acetyltransferase Hat1, Caf1 and CENP-A/H4. We show that Hat1 is required for proper CENP-A loading into chromatin, since knock-down in S2 cells leads to reduced incorporation of newly synthesized CENP-A. In addition, we demonstrate that CENP-A/Cid interacts with the HAT1 complex via an N-terminal region, which is acetylated in cytoplasmic but not in nuclear CENP-A. Since Hat1 is not responsible for acetylation of CENP-A/Cid, these results suggest a histone acetyltransferase activity-independent escort function for Hat1. Thus, our results point toward intriguing analogies between the complex processing pathways of newly synthesized CENP-A and canonical histones.

## INTRODUCTION

CENP-A is a histone H3 variant that is deposited at the centromeric region of a chromosome. The special chromatin architecture of the centromere enables the assembly of the kinetochore, which is required for microtubule attachment and faithful segregation of sister chromatids during cell divisions. In light of the importance of centromeres for the propagation of genetic information, it is intriguing that the position of the centromere within the chromosome is not determined by DNA sequence in the majority of eukaryotic organisms but instead is designated by the presence of centromere protein A (CENP-A; (1–3)). Unlike canonical histones that are highly conserved throughout evolution, however, CENP-A proteins are rather divergent from other H3 variants showing only 50–60% sequence identity to H3 in the histone fold domain and a unique N-terminal tail. Moreover, CENP-A histones from different species have limited similarity to each other (4). Like other histone variants CENP-A is incorporated into chromatin in a replication-independent fashion. In flies, CENP-A/Cid loading occurs in late mitosis in syncytial embryos in which the cell cycle lacks G1 and G2 phases (5). Outside of embryogenesis, e.g. in cultured S2 cells, the peak of CENP-A/Cid loading occurs in early G1 phase (6), although a transient increase of centromeric CENP-A/Cid was observed during mitotic metaphase (6,7). Similarly, mammalian CENP-A is loaded in G1 phase (8), although the incorporation process requires the preparation or ‘licensing’ of centromeres by specific factors during telophase of mitosis (9).

The question of precisely how CENP-A is targeted to centromeres and loaded in a cell cycle-specific manner is not completely understood but multiple studies in different organisms have revealed that a complex network of factors is required to ensure timely and spatially constrained incorporation (1,10). In mammalian cells this process involves the physical preparation of centromeric chromatin for CENP-A loading during mitosis. One of the key players in this event is the hMis18 complex that consists of Mis18α, Mis18β and M18BP1 and is recruited to the centromere by the constitutive centromeric protein CENP-C in a phosphorylation dependent manner in late mitosis (9,11,12). The Mis18 complex is required for the recruitment of the HJURP/CENP-A/H4 complex to the centromere (13). In addition, the histone chaperone FACT (Facilitates Chromatin Transcription) as well as the ATP-dependent chromatin remodeling factors CHD1 and RSF localize to the centromere and might facilitate the incorporation of CENP-A or the stabilization and remodeling of newly assembled CENP-A nucleosomes (14,15). Moreover, in human and *Xenopus* systems a role for condensin in the loading and maintenance of CENP-A at centromeric chromatin was demonstrated (16,17). Although numerous studies showed that most of these factors are essential for the assembly of CENP-A in mammals and therefore crucially important for genome stability and propagation of genetic information, several of them are not conserved in all eukaryotes. For example, the CENP-A specific chaperone HJURP lacks direct homologs in insects, nematodes and fish, although a related protein, termed Scm3, is present in *Saccharomyces cerevisiae* (18). Furthermore, in the *Drosophila* genome, no homologs have been detected for the Mis18 complex, and the ATP-dependent chromatin remodeler CHD1 is not involved in CENP-A assembly (19). Instead, it has been shown that the role of HJURP in flies is carried out by the fly-specific protein CAL1 (Chromosome Alignment Specific 1; (20,21)). CAL1 depletion results in complete abolishment of CENP-A localization at centromeres, it is associated with CENP-A at the centromere as well as in soluble complexes and it can mediate the assembly of CENP-A containing nucleosomes *in vitro* (20–23). In addition to CAL1, *Drosophila* Rbap48 (also termed Caf1) was shown to interact with nonchromatin bound CENP-A/Cid and H4 and to support CENP-A/Cid assembly *in vitro* (24). However, the nature of potential different prenucleosomal CENP-A/Cid containing complexes remains unclear to date. Here, we used a biochemical approach to characterize CENP-A/Cid prenucleosomal complexes and report on the identification of the histone acetyltransferase Hat1 as new player in the processing pathway of CENP-A/Cid in *Drosophila*.

## MATERIALS AND METHODS

### Cell lines

Several *Drosophila* S2 cell lines were engineered to contain stably integrated constructs for inducible expression of CENP-A and Hat1 fusion proteins. To this end, S2 cells were either cotransfected with pMT-based expression constructs and pCoHygro (Life Technologies) or transfected with expression constructs made with a modified pMT that contains a puromycin resistance cassette, and selection of stable integration events was performed by addition of 0.75 mg/ml hygromycin B (Gibco) or 10 μg/ml puromycin (Sigma) to the culture medium for 2–4 weeks. For further culture, the concentration of puromycin was lowered to 2 μg/ml.

### Plasmid constructs

The EGFP-CENP-A construct was made by inserting the PCR-amplified open reading frame of *Drosophila* CENP-A into the NcoI/EcoRI sites of pMT (Life Technologies) in-frame with an upstream EGFP-encoding sequence. Constructs for SF-CENP-A, SNAP-CENP-A and SF-CG2051/Hat1 were made using pMT-Puro plasmid (25). For baculovirus-mediated expression of proteins, constructs for V5-CENP-A mutant protein expression, Flag-CG2051/Hat1 and Flag-Caf1 were made using the pFastBac1 vector (Life Technologies) as a backbone. Bacterial expression constructs of Flag-CG2051 and His-CENP-A were made in pET28b (New England Biolabs). Detailed procedure in Supplementary Information.

### Protein extracts and affinity purification of CENP-A and CG2051 complexes from S2 cells

Expression of tagged proteins was induced for 24 h in stably transfected S2 cell lines using 0.5 mM (CG2051) and 5 μM (CENP-A), respectively, CuSO_4_. Cells were lysed and fractionated into cytoplasmic, nuclear and chromatin extracts. Tagged proteins were purified from S2 cell extracts (nuclear and cytoplasmic extract) by either α-GFP affinity purification or using Strep-Tactin Superflow resin followed by α-Flag affinity purification. Eluates were processed for mass spectrometry analysis. Detailed procedure in Supplementary Information.

### Mass spectrometry and data processing

For the identification of interaction partners, affinity purified CENP-A or CG2051/Hat1 samples were digested by trypsin; for analysis of post-translational modifications, CENP-A protein bands were excised from a sodium dodecyl sulphate (SDS) polyacrylamide gel and digested with trypsin or LysC as previously described (26). Resulting peptides were analyzed by reversed phase C18 HPLC coupled to a LTQ Orbitrap XL mass spectrometer (Thermo Scientific) or a Q Exactive Plus mass spectrometer (Thermo Scientific) equipped with a Nanospray Flex ionization source. Data analysis was performed using Proteome Discoverer 1.3 (Thermo Scientific) with search engine Sequest. Peptide identifications were filtered at 1% false discovery rate. Detailed procedure in Supplementary Information.

### Co-immunoprecipitation and purification of recombinant proteins

Recombinant full length V5-tagged CENP-A and truncated polypeptides, Flag-tagged CG2051 and Caf1 were expressed in *Sf*9 cells using the baculovirus system. Virus generation and amplification, protein expression and Flag-affinity purification were performed as described before (27) with minor modifications (see Supplementary Information). For protein interaction studies *Sf*9 cells were co-infected with equal amounts of the respective baculovirus stocks before affinity purification and immunoblotting. For protein interaction studies and HAT assay, respectively, His-tagged CENP-A and SF-CG2051/Hat1 were expressed in bacteria and mixtures of SF-CG2051/Hat1 and His-CENP-A extracts were purified by Ni^2+^-nitrilotriacetic acid (NTA) affinity chromatography (see Supplementary Information for details). The following antibodies were used for immunoblotting: α-CENP-A (CID) (1:2000; ab10887Abcam), α-Flag (1:10 000; F1804 Sigma), α-V5 (1:10 000; ab27671 Abcam), α-Caf1 (1:50 000; (28)).

### Size exclusion chromatography

Nuclear extracts were subjected to SEC on a 100 ml Superose 6 gelfiltration column (GE Healthcare) in Buffer A (30 mM Tris/HCl pH 8, 200 mM NaCl, 2 mM EDTA, 10% glycerol, 0.2 mM PMSF) on an ÄKTAexplorer FPLC System (GE Healthcare). Fractions of 2 ml were collected and aliquots were analyzed by western blotting using the following primary antibodies: α-CENP-A (CID) (1:2000; ab10887Abcam), α-Flag (1:10 000; F1804 Sigma), α-CAL1 (1:10 000; (21) gift of Dr A. Straight), α-Dre4 (1:1000), α-Ssrp1 (1:2000) and α-Caf1 (1:50 000; (28); gifts of Dr J.T. Kadonaga), α-H4K5ac (1:1000; NB21–2024 Upstate).

### Immunostaining of S2 cells

Immunostainings and immunofluorescence microscopy were performed essentially as described in (19). For CG2051/Hat1-CENP-A double staining, cells were incubated in 0.1% Triton X-100/PBS for 2 min before fixation to remove excess soluble Hat1. The following primary antibodies were used: α-CENP-A (CID) (1:500; ab10887Abcam); α-EGFP (1:1000; NB600–597 Novus), α-Flag (1:1000; F1804 Sigma). Specificity of the Flag antibody was determined by immunostaining of nontransfected S2 cells (Supplementary Figure S2B). After confocal microscopy, series of optical sections along the z-axis were obtained and optical sections containing CENP-A signals were projected in all channels to determine potential colocalization of CENP-A and CG2051/Hat1 (Figure 3C).

### RNAi-mediated depletion of CG2051

A dsRNA probe was generated spanning nt 71–541 of *Drosophila* CG2051 coding sequence or nt 48–573 of the bacterial Tet repressor coding sequence (negative control). Probe generation and S2 cell treatment with the dsRNA probes was performed for 4 days (Supplementary Figure S6) or 6 days (Figure 7, Supplementary Figure S6) as described previously (19). Knock-down efficiency was evaluated by reverse-transcription (RT)-qPCR on the last day of treatment as described before (29).

### Quench-chase-pulse labeling of newly synthesized CENP-A

Quenching of SNAP tag activity of existing SNAP-CENP-A was performed by a 30 min incubation of cells with 5 μM SNAP-Cell Block (NEB). New SNAP-CENP-A production was induced by 5 μM (Figure 7, Supplementary Figure S6) or 10 μM (Supplementary Figure S6) CuSO_4_. After 48 h (Figure 7, Supplementary Figure S6) or 24 h (Supplementary Figure S6) chase, cells were pulse-labeled with 4.5 μM SNAP-Cell TMR Star (NEB) for 30 min. Nonreacted TMR Star was washed out, cells were fixed in 3.7% paraformaldehyde/0.3% Triton-X100 and nuclei were counterstained with DAPI. Confocal fluorescence microscopy was performed on a Leica TCS SP5 instrument. 3D images were reconstructed and analyzed by Imaris v5.1. Statistical significance was determined by unpaired *t*-test and Mann–Whitney test using Prism5.0 software. Detailed procedure in Supplementary Information.

### Histone acetyltransferase (HAT) assay

HAT activity was measured using an established assay (30) with the following modifications: 1 μg Hat1, 1 μg Caf1 (optional) and 0.05 μCi ^14^C-acetyl-CoA were incubated with 80 μg chicken core histones or ∼2 μg recombinant His-tagged *Drosophila* CENP-A for 60 min at 25°C in a final volume of 115 μl reaction buffer (30 mM Tris/HCl, pH 8, 250 mM NaCl, 10% glycerol). Subsequently, reactions were processed for liquid scintillation spectrophotometry or subjected to 15% sodium dodecylsulphate-polyacrylamide gel electrophoresis (SDS-PAGE). After Coommassie staining the gel was dried under vacuum and exposed to a phosphoimaging screen (Fuji), which was subsequently scanned with a STORM phosphoimager system (Molecular Dynamics).

## RESULTS

### Identification of interaction partners of soluble CENP-A

**Figure 1.**
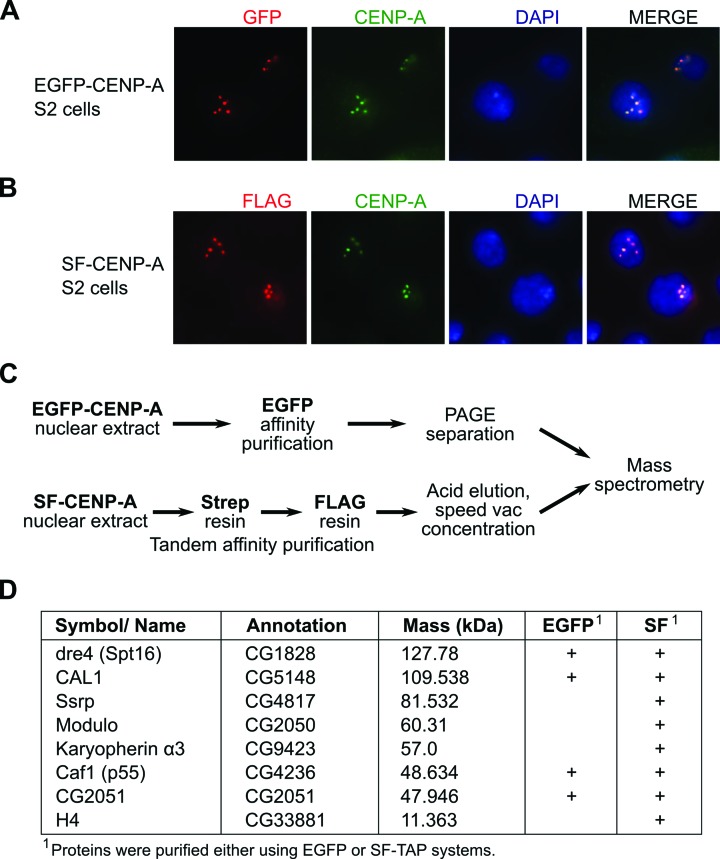
Affinity purification of CENP-A by two different strategies. (**A**and **B**) Localization of EGFP- or Strep-Flag (SF)-tagged CENP-A to centromeres in stably transfected S2 cells. Cells were stained with antibodies against GFP (A; red), Flag (B; red), CENP-A (green) and DNA was visualized by DAPI staining (blue). (**C**) Experimental scheme of the affinity purifications. (**D**) A selection of proteins identified as CENP-A interaction partners in one or both purification strategies.

To isolate protein partners of nonchromatin bound CENP-A/Cid (hereafter termed CENP-A), we used two different affinity purification strategies. We generated S2 cell lines with stable integration of either an EGFP-tagged CENP-A construct or a construct in which CENP-A was fused to an N-terminal StrepII-Flag (SF) tag. Transgene expression in both cases was controlled by an inducible metallothioneine promoter allowing fine-tuning of expression rates in order to avoid overexpression and concomitant aberrant loading of tagged CENP-A throughout the genome. Immunofluorescence staining of induced cells revealed that both CENP-A fusion proteins localized to centromeres (Figure 1A and B).

Low salt nuclear extracts were prepared from both cell lines as well as from nontransfected S2 cells and used for affinity purification by either α-GFP sepharose beads or for tandem purification by streptactin-agarose followed by α-Flag magnetic beads (SF-TAP) to identify copurifying proteins (Figure 1C). Several identical proteins were detected in eluates from both purifications indicating that they are specific interactors of soluble CENP-A (Figure 1D). Among these were *Drosophila*-specific protein CAL1 that has previously been shown to be a chaperone of *Drosophila* CENP-A (20,21), nucleolar protein Modulo that was demonstrated as a CAL1 interaction partner (31), histone chaperones Caf1 (Rbap46/48) and FACT (subunit Dre4), and CG2051. Caf1 had also been identified as a CENP-A interacting protein in *Drosophila* in a previous study (32), and FACT-Dre4 had been shown to partner CENP-A at centromeres of chicken DT4 cells (33), but has not yet been detected in soluble CENP-A complexes. In addition, histone H4, the Ssrp subunit of FACT and importin karyopherin α3 (Kap-α3) were found in our SF-TAP purification (Figure 1D). The fact that several of these proteins had previously been linked to CENP-A demonstrates that our purification strategy was specific. One protein that caught our attention was CG2051. Based on sequence comparison, CG2051 appears to be the *Drosophila* homolog of the widely conserved histone acetyltransferase 1 (Hat1; Supplementary Figure S1), which is known for its role in the acetylation of newly synthesized histone H4 (34).

### Soluble CENP-A is present in several distinct complexes

**Figure 2.**
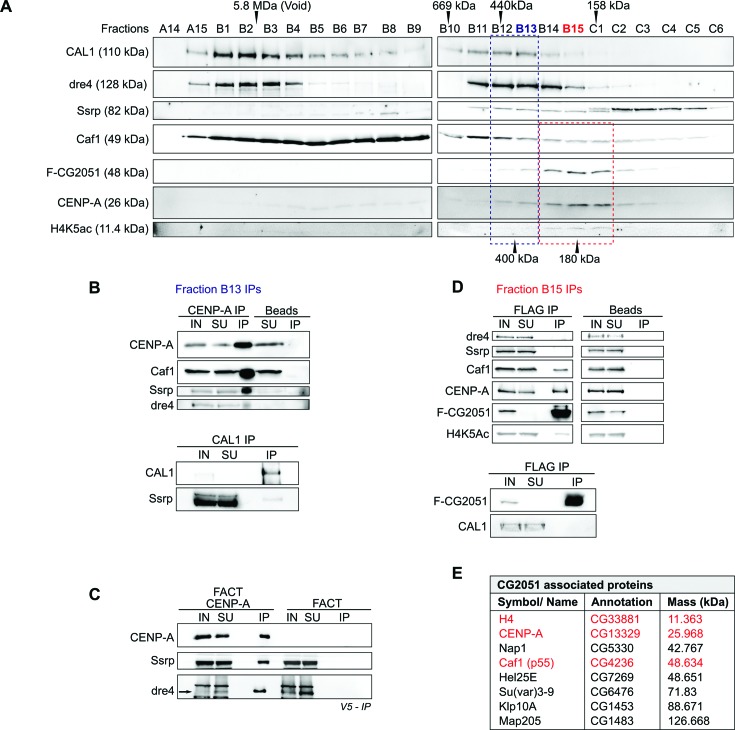
Separation of distinct prenucleosomal CENP-A containing complexes. (**A**) Nuclear extracts from S2 cells stably expressing SF-CG2051 were separated by Superose 6 size exclusion chromatography, and fractions were subjected to western blot with antibodies against the indicated proteins. Positions of several molecular mass marker proteins are indicated on top. The approximate molecular masses of the two major CENP-A containing complexes are indicated at the bottom. (**B**) Fraction B13 from (A) was subjected to immunoprecipitation (IP) with antibodies against CENP-A (upper panel) and CAL1 (lower panel). Coprecipitating proteins were detected by western blotting. (**C**) V5-pull down using extracts of *Sf*9 cells overexpressing V5-CENP-A and untagged FACT subunits Ssrp and Dre4 (left). As a control, V5-pull down was performed with extracts containing overexpressed untagged FACT only (right). (**D**) Fraction B15 from (A) was subjected to IP with antibodies against Flag-CG2051. Coprecipitating proteins were detected by western blotting. IN, input; SU, supernatant; IP, immunoprecipitate. (**D**) Selected proteins identified as interaction partners of CG2051 in a large-scale affinity purification of SF-CG2051. Proteins also detected in CENP-A purifications are highlighted in red.

To examine if CENP-A interacts with the identified proteins within a single or several distinct complexes, we performed Superose 6 size exclusion chromatography (SEC) of S2 nuclear extracts. Because no antibodies are available for *Drosophila* Hat1, we used an S2 cell line stably expressing Flag-tagged CG2051/Hat1. Immunoblotting experiments of chromatography fractions showed that the identified proteins eluted in distinct peaks (Figure 2A). Interestingly, the majority of CENP-A perfectly coeluted with CG2051/Hat1 in a complex with an apparent molecular weight (MW) of about 180 kDa. These fractions also contained the strongest signals for histone H4 acetylated at lysine 5 (H4K5ac) as well as signals for Caf1 (Figure 2A) suggesting that these proteins might form a distinct complex. In other eukaryotes, Hat1 has been shown to interact with Caf1 and the canonical histones H3 and H4 and to acetylate newly synthesized H4 at K5 and K12 (reviewed in (34)). FACT-Dre4 and CAL1 did not overlap with the CG2051/Hat1 elution pattern but instead co-eluted with each other at an apparent MW of about 400 kDa as well as in high MW fractions (> 5 MDa), although the latter showed only trace amounts of CENP-A. The 400 kDa peak also contained FACT-Ssrp. Caf1 eluted very broadly, which is consistent with its reported presence in multiple chromatin modifying complexes (35). Thus, Caf1 signals are also detected in the 400 kDa CAL1-FACT peak along with signals for CENP-A and H4. Therefore, in addition to the CG2051/Hat1 complex, prenucleosomal CENP-A forms at least one separate complex together with H4, FACT and possibly CAL1 (Figure 2A).

### CG2051/Hat1-CENP-A complex does not contain CAL1 or FACT

To corroborate these results, we performed immunoprecipitation (IP) experiments with fractions B13 (FACT-CAL1 peak) and B15 (CG2051/Hat1 peak). CENP-A antibodies pulled down FACT (Ssrp and Dre4) as well as Caf1 from Fraction B13 confirming the interaction of CENP-A with FACT (Figure 2B, upper panel). Likewise, baculovirus-mediated coexpression of V5-tagged CENP-A and untagged FACT (Dre4/Ssrp) in *Sf*9 cells resulted in coprecipitation of all three proteins upon α-V5 IP (Figure 2C). Thus, *Drosophila* CENP-A forms a stable soluble complex with FACT. IP with an antibody against CAL1 revealed that FACT-Ssrp interacts with CAL1 only weakly (Figure 2B, lower panel). This observation along with the slightly shifted elution profiles of CAL1 and FACT on SEC suggests that they are members of different CENP-A complexes.

On the other hand, antibodies against Flag-CG2051/Hat1 coimmunoprecipitated CENP-A, H4K5ac and Caf1, but not FACT or CAL1, from SEC fraction B15 (Figure 2D). To further confirm these results, we performed a large-scale immunopurification of Flag-CG2051/Hat1 from S2 cells and analyzed purified proteins by MS. Consistent with the above described results, CENP-A, Caf1 as well as H4 were detected in the purified CG2051/Hat1 sample, whereas no peptides were found for either FACT or CAL1 (Figure 2E). Together, these results demonstrate that CENP-A forms at least three distinct stable preloading complexes, one of which contains CG2051/Hat1 while the others contain either FACT or CAL1.

### Subcellular distribution of CG2051/Hat1-CENP-A complexes

**Figure 3.**
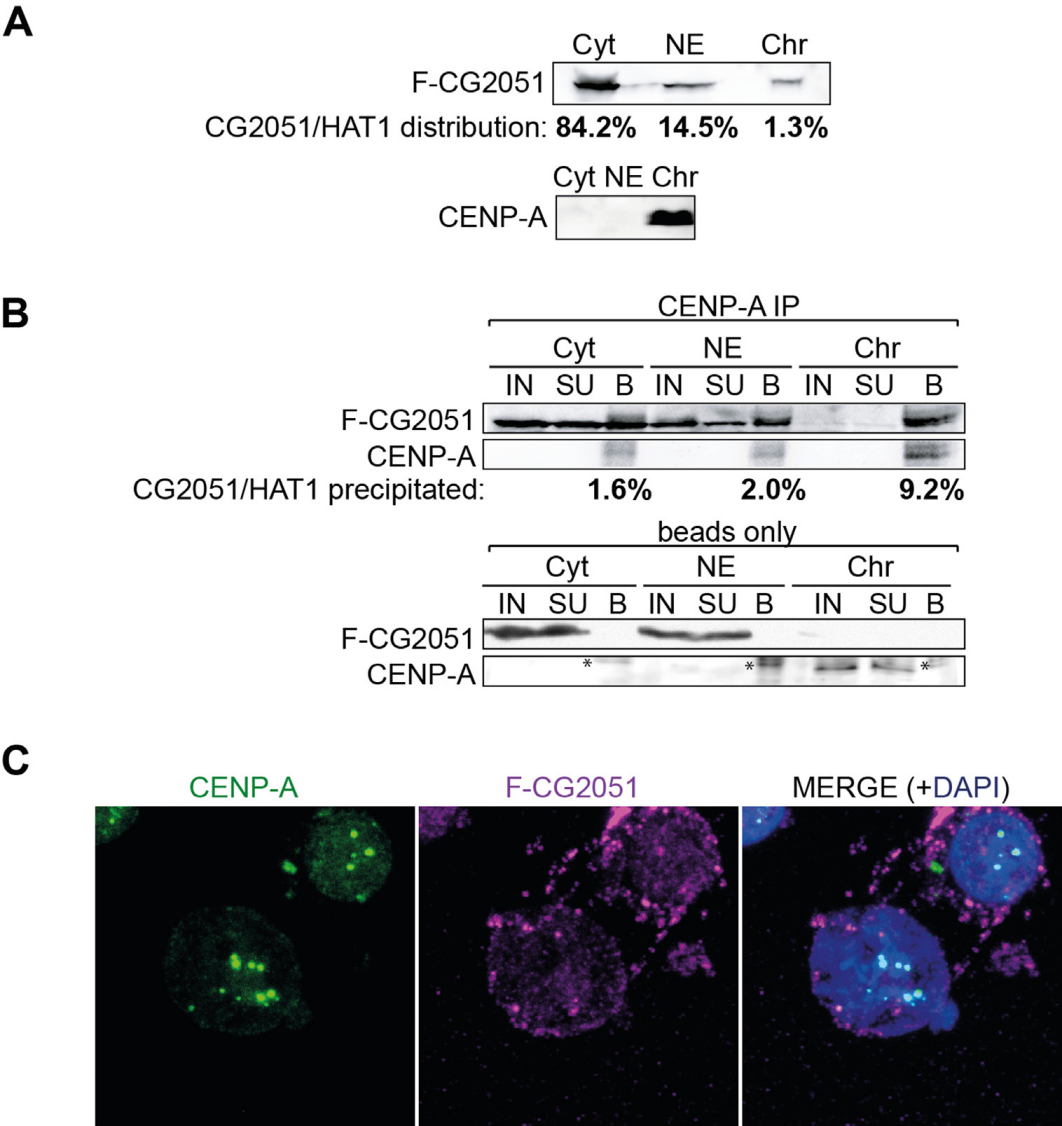
CENP-A-CG2051/Hat1 complexes are present in cytoplasm, nuclear extract and chromatin extract but the proteins do not colocalize on centromeres. (**A**) Immunoblot of cytoplasmic (*Cyt*), nuclear (*NE*) and chromatin (*Chr*) extracts probed with α-Flag (CG2051/Hat1) and α-CENP-A antibodies. CG2051/Hat1 bands were quantified and the proportion in each cellular fraction was calculated from loading volume and total extract volume. CENP-A was only detectable in *Chr*. (**B**) Immunoprecipitation of CENP-A-CG2051/Hat1 complexes was performed with α-CENP-A antibodies (upper panels) or beads only (lower panels) and subsequent western blots were probed with α-Flag (CG2051/Hat1) and α-CENP-A antibodies. Signal intensities of CG2051/Hat1 bands in input ‘IN’ and beads ‘B’ lanes were quantified, and the relative amount of pulled-down CG2051/Hat1 was calculated. SU, supernatant. Asterisks in the lower panel indicate unspecific signals in the beads fraction. (**C**) Co-immunostaining of SF-CG2051/Hat1-expressing S2 cells with antibodies against CENP-A and Flag. To remove soluble protein, cells were treated with 0.1% Triton-X100 prior to fixation. Z-projections of all planes with detectable CENP-A signal were generated from confocal images. Two representative cells are shown.

During the preparation of this manuscript, Barth *et al*. reported that CG2051 copurified with CENP-A derived from micrococcal nuclease-digested chromatin and that a tagged fusion protein colocalized with CENP-A on interphase centromeres in S2 cells (36). To determine the subcellular distribution of CG2051/Hat1-CENP-A complexes, we performed cellular fractionation experiments coupled to co-IP. Immunoblots with antibodies against marker proteins were performed to demonstrate the quality of the fractionation (Supplementary Figure S2A). Western blots of cellular fractions revealed highest abundance of Hat1 in the cytoplasm followed by nuclear extract and chromatin extract. Conversely, CENP-A was present in the chromatin extract but undetectable in nuclear and cytoplasmic extract (Figure 3A). Nevertheless, we could immunoprecipitate CENP-A from all three extracts and found that a small portion of total Hat1 amount (1.6 – 9.2%) was always associated with CENP-A (Figure 3B). Using immunostaining of S2 cells with antibodies recognizing SF-CG2051/Hat1 and CENP-A, however, we never detected colocalization of SF-CG2051/Hat1 with CENP-A at centromeres in either interphase or mitotic cells, although a portion of Hat1 was associated with chromatin (Figure 3C). We therefore conclude that CG2051/Hat1 forms stable complexes with CENP-A in nuclear and cytoplasmic extracts, yet its association with CENP-A at centromeres must be weak or transient.

### Analysis of the CENP-A/Hat1 complex

**Figure 4.**
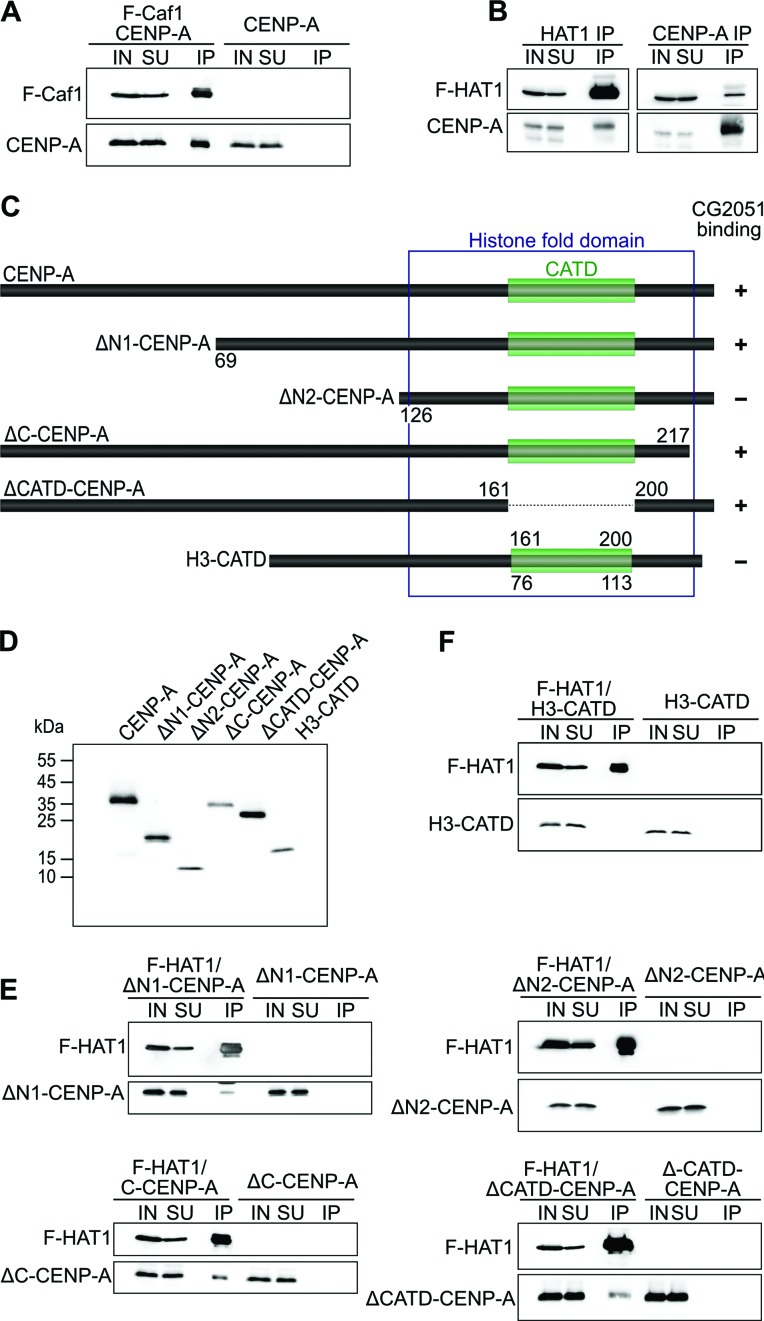
CG2051/Hat1 interacts with CENP-A directly and independently of Caf1. (**A**) Flag-pull down and subsequent immunoblotting using extracts of *Sf*9 cells overexpressing Flag-Caf1 and V5-CENP-A (left) or V5-CENP-A only (right). (**B**) Reciprocal immunoprecipitation (IP) of CG2051/Hat1-CENP-A complexes from extracts of *Sf*9 cells coexpressing Flag-CG2051/Hat1 and V5-CENP-A using either antibodies against Flag (left) or antibodies against CENP-A (right). IN, input; SU, supernatant; IP, immunoprecipitate. (**C**) Schematic representation of full length CENP-A, mutated CENP-A proteins and chimeric H3 used for interaction studies with CG2051/Hat1. The histone fold domain is highlighted by a blue frame; the CATD domain is marked as green box. (**D**) The proteins depicted in (C) were overexpressed using the baculovirus system with an N-terminal V5 tag and subjected to western blotting and immunodetection by α-V5 antibody. (**E**) Flag pull-down experiments with CG2051 and different CENP-A mutant proteins. Full length CENP-A or any of the mutated CENP-A polypeptides were co-expressed with SF-CG2051 in *Sf*9 cells, immunoprecipitated with α-Flag resin and subjected to immunoblotting with α-Flag and α-V5 antibodies. Control IPs were performed with extracts from cells expressing the respective CENP-A mutant proteins but no SF-CG2051. (**F**) Flag pull-down experiments with CG2051 and the H3-CATD chimeric protein. Experiment was done as in (E).

In *S. cerevisiae* the HAT1 acetyltransferase core complex consists of the enzyme Hat1p and the chaperone Hat2p (also termed Rbap46/48, Caf1-p55, Caf1 in other organisms;(37)). This complex appears to be conserved across species, as it has been detected in all organisms in which Hat1 has been studied to date (reviewed in (34)). Caf1 (Rbap48) had previously been found to interact with soluble *Drosophila* CENP-A (24). The CENP-A interacting CG2051/Hat1 containing complex that we have identified by affinity purification and SEC also contained Caf1 (Figure 2A and D). Thus, it was possible that Caf1 rather than Hat1 is the direct interaction partner of CENP-A in this complex. To distinguish between these possibilities, we performed co-IP experiments with baculovirus-generated recombinant proteins. When we co-expressed Flag-tagged Caf1 with V5-tagged CENP-A a robust interaction between these proteins was detected upon Flag-IP while no coprecipitation occurred in extracts containing only CENP-A (Figure 4A) consistent with previous results (24).

We also found that co-expression of Flag-CG2051/Hat1 and V5-CENP-A and subsequent IP with either α-Flag or α-V5 antibodies resulted in clear mutual co-IP of both proteins (Figure 4B). As an additional control, we performed western blots of fractions derived from the Flag-copurification and incubated them with antibodies against Caf1 and H4. This experiment showed the presence of a signal slightly above a 55 kDa protein size marker, which is similar to the electrophoretic mobility of *Drosophila* Caf1 and therefore likely corresponds to endogenous Caf1 from *Sf*9 cells (*Sf*9 Caf1 sequence is unknown to date; Supplementary Figure S3) while no signal was detected for H4 (data not shown). Note, however, that the same amount of sample was used for Coommassie gel and western blot suggesting that the relative portion of endogenous Caf1 might be small. To further elucidate the exact nature of the interaction between CG2051 and CENP-A we expressed His-tagged CENP-A and Flag-CG2051/Hat1 in *Escherichia coli* and copurified them by His-tag-specific Ni^2+^-affinity chromatography. Although Flag-CG2051/Hat1 copurified with His-CENP-A, further controls showed that Flag-CG2051/Hat1 also bound to the Ni^2+^-beads in the absence of His-CENP-A (data not shown). In summary, although our data point toward a direct interaction between CG2051/Hat1 and CENP-A, at this point we cannot conclusively answer this question.

### CENP-A interacts with HAT1 via an N-terminal region

CENP-A proteins from different organisms are quite diverse with respect to their primary structure, yet a region within the histone fold domain termed the CATD domain exhibits a slightly higher degree of conservation, and it has been shown to determine the specific targeting of CENP-A to the centromere (38,39). The CATD from mammalian CENP-A has previously been found to direct the interaction with the CENP-A specific chaperone HJURP (40). To investigate whether the CATD of *Drosophila* CENP-A holds a similar function in the interaction with CG2051/Hat1, we expressed a panel of V5-tagged CENP-A mutated polypeptides in *Sf*9 cells (Figure 4C and D) and performed Flag-pull-down experiments with recombinant Flag-CG2051/Hat1 (Figure 4E). Deletion of the CATD (amino acids 162–199) had no effect on the interaction between CENP-A and CG2051/Hat1, indicating that the CATD is not involved in CG2051/Hat1 interaction. This was further confirmed by the failure of CG2051/Hat1 to pull down a chimeric histone H3 protein containing the CENP-A CATD (Figure 4F). Also, the deletion of a small C-terminal region of CENP-A had no effect on CG2051/Hat1 interaction. In contrast, when we deleted the N-terminal 125 amino acids of CENP-A, the interaction with CG2051/Hat1 was completely abolished. Using another N-terminally truncated protein that lacks residues 1–68, we delimited the region required for interaction with CG2051/Hat1 to residues 69–125 (Figure 4C and E). Although we cannot completely rule out the involvement of endogenous *Sf*9 Caf1 (Supplementary Figure S3), the data suggest that CG2051/Hat1 interacts with *Drosophila* CENP-A via a region adjacent to the histone fold domain while it does not require the CATD, the very N-terminal or the C-terminal region for this interaction.

### Posttranslational modifications of *Drosophila* CENP-A

**Figure 5.**
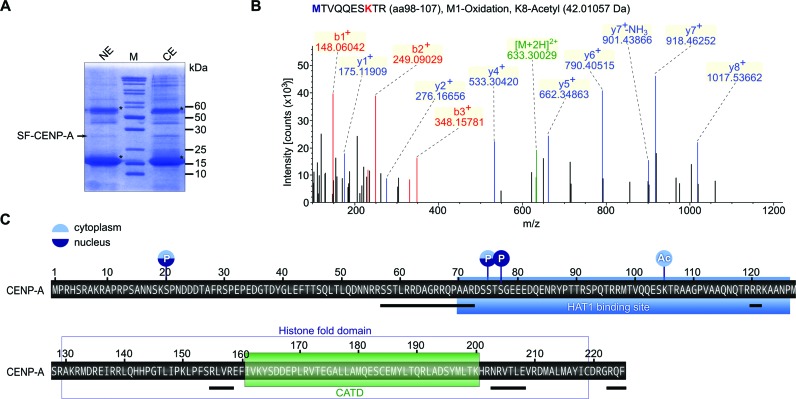
Prenucleosomal CENP-A is post-translationally modified by acetylation and phosphorylation. (**A**) Coommassie-stained SDS gel showing SF-CENP-A purified from nuclear (NE) or cytoplasmic (CE) extracts of S2 cells. Asterisks designate IgG heavy and light chains. (**B**) MS spectrum of a peptide obtained by trypsin digestion of cytoplasmic CENP-A showing acetylation of lysine 8, which corresponds to lysine 105 in full length CENP-A. The peptide sequence is shown at the top. (**C**) Schematic depiction of the positions of detected modifications in cytoplasmic (light blue) or nucleoplasmic (dark blue) CENP-A. P, phosphorylation; Ac, acetylation. The CG2051/Hat1 interaction domain is marked with a blue box and the CATD domain with a green box. Black lines below the sequence delineate peptides that were not covered by MS analysis.

Given the physical link between the potential HAT CG2051 and CENP-A, we next asked if soluble CENP-A is acetylated *in vivo*. To this end, we analyzed the post-translational modification pattern of CENP-A purified from cytoplasmic or nuclear extracts of S2 cells (Figure 5A). CENP-A from both samples was digested with trypsin or LysC, and peptides were analyzed by high-resolution electrospray ionization mass spectrometry (ESI-MS). By this approach a total of 87.6% of the CENP-A sequence was interrogated including all lysine residues (Figure 5C). We identified lysine 105 (K105) to be acetylated in the cytosolic CENP-A sample (Figure 5B). In CENP-A derived from nuclear extracts, we found peptides corresponding to nonacetylated K105 but none that contained K105ac indicating that *Drosophila* CENP-A is indeed acetylated and that acetylation might depend on the localization of CENP-A.

In addition to K105ac, we found phosphorylation of serines 20 and 75 in both cytoplasmic and nucleoplasmic CENP-A and of serine 77 in nucleoplasmic CENP-A (Figure 5C and Supplementary Figure S4). Although S75 and S77 lie within a tryptic peptide spanning a sequence with several other serines (residues 73–91, Figure 5C), the use of high-resolution MS allowed for unambiguous assignment of phosphorylated residues. Of note, this peptide was found in unmodified, mono- and diphosphorylated form in the nucleoplasmic CENP-A sample, while we only detected the unmodified and monophosphorylated (S75ph) peptide in the cytoplasmic sample. Along with the differential K105 acetylation, these results suggest distinct post-translational modification patterns in different preloading states of *Drosophila* CENP-A.

### CG2051 is the *Drosophila* homolog of Hat1 with specificity toward H4

**Figure 6.**
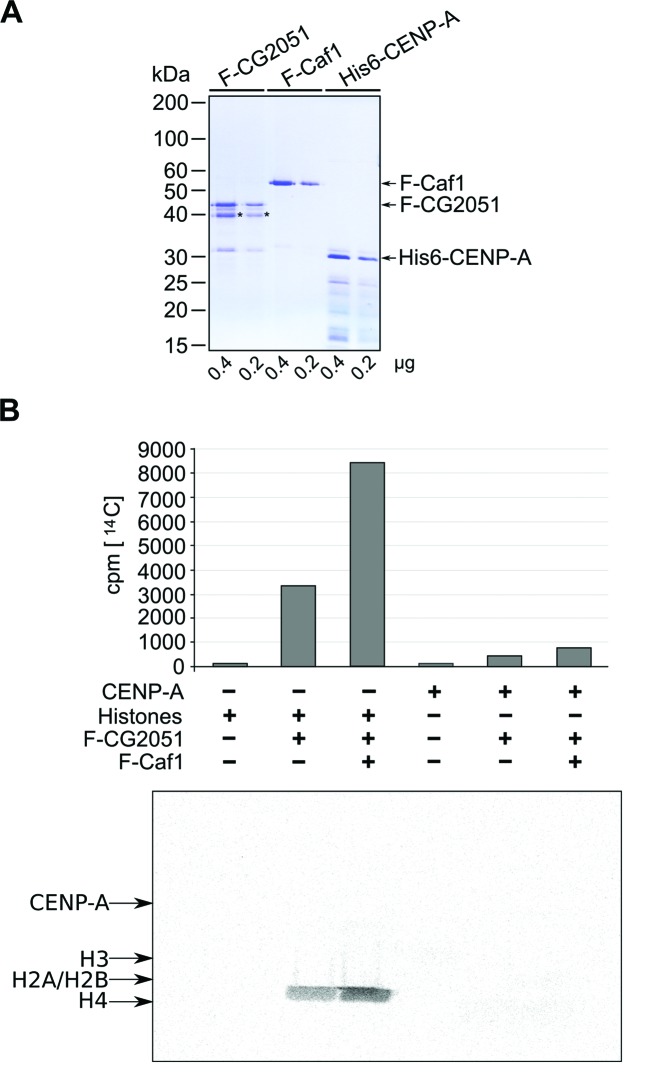
CG2051 is the enzymatically active homolog of Hat1 in *Drosophila*. (**A**) SF-CG2051/Hat1 and SF-Caf1 were overexpressed in *Sf*9 cells and purified by Flag-affinity purification. Full length CENP-A was expressed as a His-tagged protein in bacteria and purified via Ni^2+^-NTA chromatography. 0.4 and 0.2 μg of each protein were separated by SDS-PAGE and detected by Coommassie staining. Asterisks denote a contaminating band or a degradation product in the CG2051 preparation. (**B**) HAT assay with recombinant CG2051 and either core histones or *Drosophila* CENP-A as a substrate. After incubation in the presence of ^14^C-acetyl-CoA, half of the reaction mixture was subjected to liquid scintillation counting (upper panel). The other half was separated by 16% SDS-PAGE, stained with Coommassie brilliant blue and processed for autoradiography (lower panel). Positions of histones were determined by aligning the autoradiogram with the stained gel.

The fact that CENP-A is acetylated and that the location of the acetylated lysine overlaps with the binding region of the potential HAT CG2051/Hat1 prompted us to examine the possibility that CENP-A might be a substrate for Hat1. The only known substrate for Hat1 to date is histone H4, which is acetylated at lysines 5 and 12 (K5 and K12) by this enzyme (34). Therefore, we first established that *Drosophila* CG2051/Hat1 indeed is a histone acetyltransferase using purified recombinant proteins (Figure 6A) for *in vitro* HAT assays. A liquid scintillation counting-based assay revealed significant HAT activity of Flag-Hat1 (Figure 6B upper panel), and autoradiography of separated histones demonstrated its specificity toward histone H4 (Figure 6B, lower panel). In keeping with findings from other species (37,41), Hat1 activity was further stimulated by the addition of recombinant Caf1 (Figure 6B). We then tested Hat1 activity toward CENP-A as a substrate. Full length His-tagged CENP-A was expressed in bacteria and purified using Ni^2+−^affinity chromatography (Figure 6A). Weak HAT activity was detected when Hat1 was incubated with His-CENP-A, and this activity was slightly enhanced by addition of Caf1 (Figure 6B). However, no signals were detected by autoradiography.

To further clarify whether Hat1 is able to modify CENP-A, we performed HAT assays with short peptides corresponding either to the N-terminal tail of H4 or to residues 2–18 (containing a K within an RAKR motif that occurs twice within the CENP-A sequence) and residues 97–113 (containing K105 that is acetylated *in vivo*, Figure 6B), respectively, of CENP-A. These experiments revealed that Hat1, although highly active toward the H4 control peptide, did not modify any of the CENP-A peptides (Supplementary Figure S5). Together these data establish CG2051 as the *Drosophila* ortholog of yeast and vertebrate Hat1. In addition, they provide evidence for a role of Hat1 as an escort for preloading CENP-A that is independent of its function as a HAT.

### The effect of Hat1 depletion on CENP-A loading

**Figure 7.**
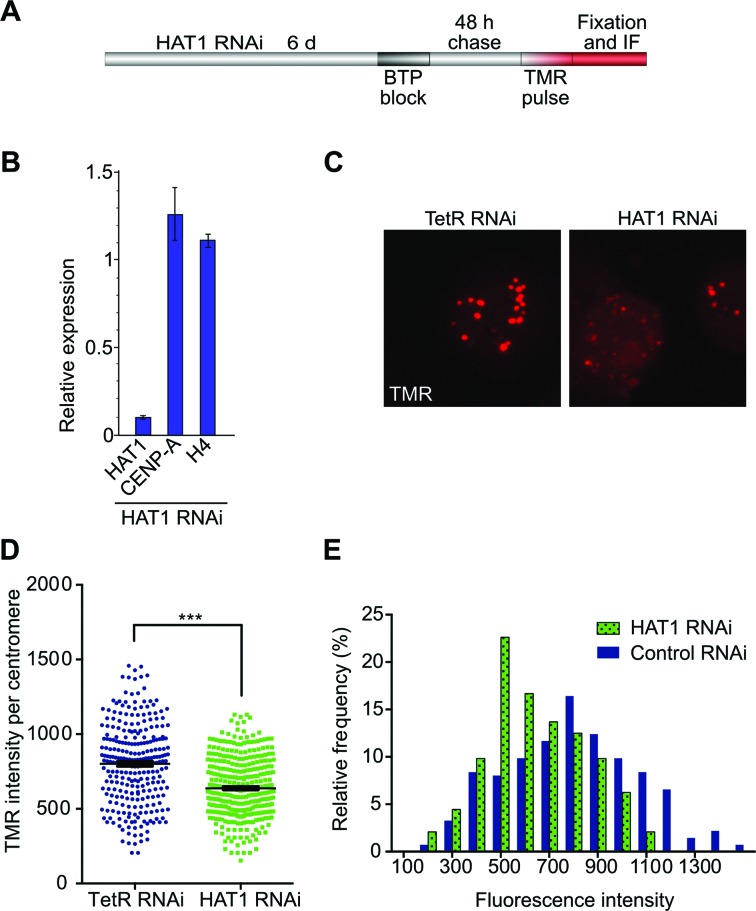
Hat1 knock-down causes decreased CENP-A loading. (**A**) Experimental design of pulse labeling-mediated analysis of CENP-A centromeric loading. (**B**) RNAi efficiency was determined by RT-qPCR for Hat1. Transcript levels for Hat1, CENP-A and histone H4 in Hat1 RNAi cells were normalized against GAPDH and are expressed relative to those of the respective genes in TetR-treated S2 cells (control). (**C**) Example image of reduced CENP-A intensities in Hat1 RNAi cells. Cells were processed according to the scheme in (A) and newly loaded SNAP-CENP-A was visualized by staining with TMR-Star. Images were acquired and processed with identical settings. (**D**) Quantification of SNAP-CENP-A intensities in Hat1 knock-down and TetR RNAi control cells using Imaris v5.1 software. Statistical significance was determined by unpaired *t*-test and Mann–Whitney test (***P < 0.0001). (**E**) Histogram showing the distribution in intensity of centromeric SNAP-CENP-A signals in Hat1 RNAi and control cells.

Next we investigated if Hat1 affects the loading of CENP-A onto centromeric chromatin. To monitor the incorporation of newly synthesized CENP-A, we used a quench-chase-pulse labeling technique that relies on a SNAP-tag fused to CENP-A (8,42). We generated stable S2 cell lines allowing for controlled expression of SNAP-CENP-A. Low concentrations of CuSO_4_ led to mild induction and centromere-specific localization of SNAP-CENP-A as shown in labeling experiments with tretramethylrhodamine (TMR)-linked SNAP substrate (TMR-Star; Figure 7C). To monitor the effects of Hat1 on SNAP-CENP-A loading, we depleted Hat1 by RNAi. Due to the lack of suitable antibodies against Hat1, RNAi efficiency was validated by qPCR. In addition, we examined if Hat1 depletion affects transcription of the CENP-A and H4 genes, respectively. Cells treated with Hat1 dsRNA showed 90% reduction in Hat1 mRNA compared to control cells (treated with dsRNA against Tet Repressor), while CENP-A and H4 transcript levels were unaffected (Figure 7B).

Four or six days, respectively, after Hat1 dsRNAi treatment, the nonfluorescent SNAP substrate was added to the cells to block the SNAP-tags of all existing CENP-A molecules. Following a 24 or 48 h chase period, labeling of newly synthesized SNAP-CENP-A was performed by addition of TMR-Star (Figure 7A and C). Fluorescence intensity measurements and quantification revealed a highly significant decrease in newly incorporated CENP-A upon Hat1 knock-down (*P* < 0.0001). The extent of decrease was in the range of 20–43% in three independent experiments (Figure 7D and Supplementary Figure S6). A distribution analysis of the TMR intensities of individual centromeres revealed accumulation of lower intensity spots in Hat1 RNAi cells compared to control cells. (Figure 7E and Supplementary Figure S6).

Since Hat1 is known to be involved in DNA replication through the H3-H4 loading pathway, we also determined the cell cycle profiles of dsRNAi-treated cells in two experiments to rule out indirect effects on CENP-A loading due to a cell cycle block in S-phase. However, no significant differences were detected between Hat1 RNAi and control cells (Supplementary Figure S7). Taken together, our data strongly suggest that Hat1 is physically and likely functionally linked to the CENP-A assembly pathway in *Drosophila*.

## DISCUSSION

### Multiple CENP-A containing preloading complexes

The machinery involved in delivering and preparing newly synthesized CENP-A for loading to centromeric chromatin is only partially understood in *Drosophila*. Here, we provide evidence for the existence of multiple distinct complexes that bind to prenucleosomal CENP-A. Two of these complexes contain the chaperone proteins FACT or CAL1, respectively, while the third reveals a stable association of CENP-A with the histone acetyltransferase Hat1 and its binding partner Caf1. Previous studies have identified the *Drosophila*-specific protein CAL1 as the major CENP-A chaperone showing that ablation of CAL1 leads to a loss of CENP-A incorporation at the centromeres (20–23). Our affinity purification and SEC experiments confirm these observations and in addition show that the major CAL1-CENP-A containing complex has a molecular mass of ∼400 kDa suggesting the presence of additional proteins. However, although the two subunits of the histone chaperone FACT coelute with CAL1 on the SEC column, only small amounts of Ssrp coprecipitate with CAL1 in these fractions suggesting that CAL1 forms a complex lacking FACT. The majority of CAL1, however, seems to be contained in a larger complex eluting in the void volume of the SEC column similar to the Dre4 but not the Ssrp subunit of FACT. These fractions contain only trace amounts of CENP-A suggesting a CENP-A-independent CAL1 complex. It has been shown before that a large fraction of CAL1 protein in the cell does not colocalize with CENP-A (23), thus giving support to the notion that CAL1 might be present in a complex lacking CENP-A.

The FACT complex has been identified in human CENP-A complexes purified from nucleosomal extracts (43,44), and it was shown to colocalize at centromeres and to be involved in CENP-A loading in chicken DT40 cells (33). Our data now also provide evidence for a nonchromatin associated FACT-CENP-A complex in *Drosophila*. Of note, the Ssrp subunit of FACT, which is known to be targeted by phosphorylation (45), is likely to be in a hypo- or unphosphorylated state within this complex, while monomeric Ssrp appears as a slightly larger band on western blot indicating phosphorylation (Figure 2A).

### Hat1 and prenucleosomal CENP-A

Interestingly, our data also demonstrate that the majority of prenucleosomal CENP-A is present in a complex of ∼180 kDa containing the *Drosophila* homolog of histone acetyltransferase Hat1, the chaperone Caf1 and histone H4 but not FACT or CAL1 (Figure 2). The HAT1 complex, consisting of Hat1 and Hat2 (also termed Caf1, Rbap46/48, p55) is well known for its function in acetylating newly synthesized histone H4 at K5 and K12 (34), and consistent with that, we found that H4 present in this complex carries K5 acetylation. The CENP-A-Hat1 complex is detectable in cytoplasmic, nuclear and chromatin fractions of the cell. Biochemical fractionation is a somewhat crude way to determine subcellular localization of proteins, and it is therefore possible that CENP-A-Hat1 complexes detected on chromatin might in fact represent contaminating soluble nuclear proteins (e.g. from stray unlysed nuclei contaminating the chromatin fraction). Nevertheless, our inability to detect Hat1 on centromeres by immunostaining stands in contrast to the results from Barth *et al.*, who reported centromeric localization of Hat1 (36). It is unknown, however, whether colocalization in the previous study was found on all or only a small fraction of interphase cells/centromeres. Based on our data, we conclude that if Hat1 associates with centromeres it must be a transient event occurring only during a short window of the cell cycle.

### *Drosophila* Caf1 enhances Hat1 activity and interacts with CENP-A

Caf1 has previously been shown to interact with fly CENP-A in a ternary complex with H4 (24). It was also found to copurify with mammalian HJURP-CENP-A complexes (46), and to interact with the centromere-‘priming’ Mis18 complex (47). Here, we confirm for the *Drosophila* protein that Caf1 interacts with Hat1, that it enhances Hat1 histone acetyltransferase activity and that it binds CENP-A. In yeast and mammals, Hat1 frequently associates with another chaperone protein termed Hif1 or NASP, respectively (48–51). However, we did not detect the fly homolog of Hif1, CG8223, in either of the CENP-A purification approaches, yet CG8223 was present in the pool of proteins obtained by Hat1-affinity purification (data not shown). Hence, it is likely that the interaction between NASP and Hat1 is conserved in flies, although CG8223/NASP does not appear to be involved in the CENP-A-Hat1 complex.

### Hat1 as a chaperone of CENP-A?

It has been shown that newly synthesized human H3-H4 pass through multiple complexes on their way from the site of synthesis to their incorporation into chromatin (51). In this process Hat1 interacts with H3-H4 in the cytoplasm and acetylates H4, but remains associated or re-associates with the histones in the nucleus. Since it is unusual for an enzyme to remain bound to its substrate after completion of the catalytic reaction, it has been suggested that Hat1 might act in a chaperone-like fashion in addition to its enzymatic activity (34). Our study shows that CENP-A-H4, similar to canonical H3-H4, are part of discrete complexes prior to their assembly into centromeric chromatin, one of which contains Hat1. While H4 in this complex bears K5 acetylation and is therefore the likely substrate for Hat1, Hat1 also interacts (directly or indirectly) with CENP-A via its N-terminal domain but does not acetylate this histone. In contrast, Hat1 does not interact with H3 bearing the Cenp-A CATD. Thus, Hat1 or the Hat1/Caf1 complex might act the part of a chaperone with specificity for prenucleosomal CENP-A-H4. Alternatively, it might use the handle on CENP-A to remain associated with the complex to maintain acetylation on H4.

### Posttranslational modifications on pre-nucleosomal CENP-A

Our analyses revealed that prenucleosomal *Drosophila* CENP-A is modified by acetylation and phosphorylation. We found all modified amino acids to be located in the poorly conserved N-terminal region of the protein. Thus, it is not surprising that unlike in canonical histones the modified residues are also not conserved between different species. Acetylation was previously reported for chromatin-bound human CENP-A. However, the acetylated lysine (K124) is located in the histone fold domain close to the C-terminus and thus it is likely to have different functional consequences (52). Moreover, human CENP-A was reported to be trimethylated at the α-N position of glycine 1 and phosphorylated at two serines (S16, S18) in the N-terminal tail (53). In addition, S7 is phosphorylated during mitosis and required for mitotic progression (54–56). Since we analyzed an N-terminally tagged CENP-A we could not determine a potential α-N methylation in the *Drosophila* protein. Two of the three phosphorylation sites in *Drosophila* CENP-A are closely spaced (S75 and S77), which is reminiscent of the human pattern (S16, S18). Phosphorylation of human S16 and S18 has been suggested to affect higher order folding of CENP-A-containing nucleosomal arrays (53). However, both the length of the N-terminal region as well as the sequence context of the phosphorylation sites are quite different in flies and humans. It remains to be studied if the three phosphorylation sites in fly CENP-A are also present in chromosomal CENP-A to affect higher order folding in a similar fashion as in the human system.

Interestingly, we find that the modification state of prenucleosomal *Drosophila* CENP-A appears to be linked to its location within the cell. We found acetylation on K105 exclusively in CENP-A purified from cytoplasmic extracts but did not detect it in three independent experiments with nuclear extract (although we readily detected the unmodified residue). Thus, it is possible that CENP-A acetylation is important for its association with complex partners and/or for its import into the nucleus similar to what has been shown for H4 acetylation at K5 and K12 (57,58). Moreover, while we only detected phosphorylation at S20 and S75 in cytoplasmic CENP-A, we found additional phosphorylation of S77 in nucleoplasmic CENP-A. Changes in the modification pattern have been demonstrated for pre-nucleosomal H3 and H4 (58), although the functions of some of these modifications are not yet clear. It is possible that shifting CENP-A modification patterns may be involved in regulating the passage of newly synthesized CENP-A through different pre-assembly complexes.

### Requirement of Hat1 for Cenp-A loading in vivo

RNAi-mediated knock-down of Hat1 revealed reduced incorporation of newly synthesized CENP-A into centromeric chromatin. There are several potential explanations for this finding. The most straightforward one is that Hat1 acts directly in the CENP-A assembly pathway. Since the observed decrease in loading efficiency was moderate, it is possible that one of the other chaperone complexes can partially compensate for the lack of Hat1 upon depletion. Alternatively, the role of Hat1 may be indirect. On one hand, its known involvement in replication-dependent assembly of canonical H3-H4 may impair cell cycle progression and consequently CENP-A loading. The undisturbed cell cycle profiles of Hat1 RNAi-treated cells, however, argue against this possibility. On the other hand, depletion of Hat1 may perturb chromatin structure due to potential H3-H4 loading defects leading to transcriptional deregulation of factors directly involved in CENP-A assembly. While we have shown that transcription of CENP-A itself and H4 is not perturbed by Hat1 depletion, we cannot rule out that other assembly pathway components might be affected. In contrast to the results shown here, Barth *et al*. found no decrease in the intensity of GFP-tagged CENP-A at centromeric foci upon CG2051 RNAi (36). This may be due to methodological differences. Our quench-chase approach enables the study of newly incorporated CENP-A only, thus making smaller differences more likely to be detected. GFP-intensity measurements, on the other hand, necessarily detect pre-existing as well as newly incorporated protein and therefore might have missed the incorporation defect upon RNAi treatment. In any case, the exact nature of the functional role of Hat1 in CENP-A assembly awaits further elucidation in the future.

### Does mammalian CENP-A assembly also involve Hat1?

So far, studies on Hat1 have focussed on its well-established role in the processing pathway of newly synthesized H3-H4 dimers. Despite the high degree of conservation of the enzyme as well as of the substrate sites on H4, deletion of Hat1 in yeast, chicken DT40 or mouse cells did not cause significant defects in cell proliferation. Nevertheless, it has clearly been shown that Hat1 is responsible for H4 K5/K12 acetylation *in vivo*, and Hat1 mutants display defects in telomeric silencing and DNA damage repair, both processes being sensitive to chromatin structure alterations (37,57,59–66). Recently, Nagarajan *et al*. found that Hat1 knock-out in the mouse caused perinatal lethality with developmental anomalies in the lung and skeletal defects (63). Interestingly, mouse embryonic fibroblasts (MEFs) from these mice exhibited a moderate accumulation of G2/M cell cycle stages and increased occurrence of chromosomal defects, such as chromatid breaks, aneuploidy or cells with 4n DNA content. While these defects might be related to problems with general chromatin assembly or DNA damage repair as suggested, it is tempting to speculate that CENP-A loading and thus centromere formation is compromised in these cells. Future experiments should reveal if the role of Hat1 in CENP-A assembly is conserved between flies and mammals.

## Supplementary Material

SUPPLEMENTARY DATA
