# Supplementary material for: A novel role for the histone acetyltransferase Hat1 in the CENP-A/CID assembly pathway in Drosophila melanogaster
Source: Nucleic Acids Res. 2015 Nov 19;44(5):2145–59. doi: 10.1093/nar/gkv1235 (PMC4797270; doi:10.1093/nar/gkv1235)
Supplement: SUPPLEMENTARY DATA [file supp_44_5_2145__index.html]

A novel role for the histone acetyltransferase Hat1 in the CENP-A/CID assembly pathway in Drosophila melanogaster — A novel role for the histone acetyltransferase Hat1 in the CENP-A/CID assembly pathway in Drosophila melanogaster — SUPPLEMENTARY DATA 

# A novel role for the histone acetyltransferase Hat1 in the CENP-A/CID assembly pathway in *Drosophila melanogaster*

## SUPPLEMENTARY DATA

- SUPPLEMENTARY DATA
