# Supplementary material for: A novel role for the histone acetyltransferase Hat1 in the CENP-A/CID assembly pathway in Drosophila melanogaster
Source: Nucleic Acids Res. 2015 Nov 19;44(5):2145–59. doi: 10.1093/nar/gkv1235 (PMC4797270; doi:10.1093/nar/gkv1235)
Supplement: SUPPLEMENTARY DATA [file supp_gkv1235_nar-01463-v-2015-File009.pdf]

Supplementary Information: **A novel role for the histone acetyltransferase Hat1 in the CENP-A/CID assembly pathway in *Drosophila melanogaster***

Mark Boltengagen, Anming Huang, Anastasiya Boltengagen, Lukas Trixl, Herbert Lindner, Leopold Kremser, Martin Offterdinger, Alexandra Lusser

Supplementary materials and methods

Plasmid constructs

Constructs for StrepII-Flag-CENP-A, SNAP-CENP-A and StrepII-Flag-CG2051/Hat1 were made using pMT-Puro plasmid (Iwaki and Castellino 2008). A StrepII-Flag (SF) encoding sequence was amplified by PCR with primers that introduced a *Drosophila* Kozak sequence (CAAC) upstream of the ATG start codon and the plasmid pDEST/N-SF (Gloeckner et al. 2009) as a template and cloned into the KpnI/NotI sites of pMT-Puro. Likewise, a SNAP tag was introduced into pMT-Puro using a PCR-amplified SNAP sequence including the Kozak sequence upstream of the ATG start codon. Subsequently, the CENP-A ORF was cloned into KpnI/XhoI sites of pMT-SF-Puro or pMT-SNAP-Puro, respectively, downstream of and in frame with the respective tags. The CG2051 ORF was PCR-amplified from cDNA and cloned into NotI/XhoI digested pMT-SF-Puro.

For baculovirus-mediated expression of proteins, Flag- or V5-tag encoding sequences were introduced into NotI/NdeI (V5) or SalI/NdeI (Flag) sites of pFastBac1 (Life Technologies). The ORFs of CG2051 and Caf1 were amplified from cDNA by PCR and cloned into NdeI/XhoI digested pFastBac-Flag to generate N-terminally tagged sequences. All CENP-A mutant sequences were amplified by PCR and inserted in frame with the upstream V5 tag into the NdeI/XhoI digested pFastBac-V5 vector. The CENP-A encoding ORFs span the following amino acid sequences: full length CENP-A (aa 1-225); ΔCATD-CENP-A (deletion of aa 162-199); ΔN1-CENP-A (aa 69-225); ΔN2-CENP-A (aa 126-225); ΔC-CENP-A (aa 1-217). To generate an H3-CATD hybrid protein, the H3 sequence covering aa 76-113 was replaced by the CENP-A CATD (aa 162-199).

For protein expression in *E. coli*, SF-CG2051/Hat1 was amplified by PCR from pMT-SF-CG2051 (see above) and cloned into the NcoI/XhoI digested pET28b vector (New England Biolabs). Similarly, the CENP-A coding region was amplified and cloned into the NdeI/BamHI sites of pET28b to create an in-frame fusion with an N-terminal His-tag. The plasmids were transformed into *E. coli* BL21(DE3) *Rosetta* cells.

Extract preparation and affinity purification of CENP-A and CG2051 complexes

Purification from S2 cells: Expression of tagged proteins was induced for 24 h in stably transfected S2 cell lines using 0.5 mM $CuSO_4$. All following steps were performed at 4°C. 10^9 cells were harvested, washed with PBS and lysed by addition of 10 ml ice cold Buffer L (10 mM Tris/HCl, pH 8, 340 mM sucrose, 10 mM KCl, 3 mM $CaCl_2$, 2 mM $MgCl_2$, 0.1 mM EDTA, 1 mM DTT, 0.2 mM PMSF, 0.5 mM benzamidine) to the cell pellet followed by douncing in a glass douncer for 2-3 min. The cell suspension was incubated on ice for 10 min before douncing was repeated. Nuclei were collected by centrifugation at 3700 g for 15 min, mixed with 5 ml Buffer N (30 mM HEPES/KOH, pH 7.9, 150 mM NaCl, 1.5 mM $MgCl_2$, 0.05% NP-40, 10% glycerol, 0.2 mM EDTA, 0.5 mM DTT, 1x protease inhibitor cocktail (Roche)), homogenized by douncing for 2-3 min, incubated on ice with gentle stirring for 30 min and finally centrifuged for 30 min at 20,000xg. The resulting nuclear extract was used for affinity purification of tagged CENP-A as well as for western blotting. The remaining chromatin pellet was resuspended in 1 ml Buffer N supplemented with 0.15 U/µl benzonase (Novagen) and incubated for 1 h with gentle stirring before addition of NaCl to 400 mM final concentration and additional incubation for 15 min. The chromatin extract was cleared by centrifugation as before and used for western blot analysis. The supernatant after cell lysis, which contains predominantly cytoplasmic proteins, was cleared again by ultracentrifugation for 1 h at 45,000xg in a Beckmann L-70 ultracentrifuge and used for affinity purification of SF-CENP-A and western blotting. Cell fractionation quality was determined by immunoblotting using the following antibodies against: actin (cytoplasmic marker; 1: 2000; Sigma), transcription factor Zf30C (nuclear extract marker; 1: 20000; home made), histone H3 (chromatin marker; 1: 20000, Millipore).

For purification of EGFP-CENP-A complexes, nuclear extract was precleared with protein A-sepharose beads (1:1 slurry) equilibrated in Buffer N before 3 µg of anti-GFP antibody (Novus Biological) per 1 ml extract was added and incubated overnight with rotation. 100 µl of Protein A sepharose slurry were added, incubated for 2 h and centrifuged for 5 min at 2000xg. Beads were extensively washed with Buffer N and finally eluted with 100 mM citric acid (pH 3) for 2 min. Eluted proteins were precipitated with 20% trichloroacetic acid/1 mg/ml deoxycholate, redissolved in 1x Lämmli sample buffer and subjected to SDS-PAGE and Coomassie staining. Gel lanes were cut into 5 equal pieces for further in-gel protease digestion and mass spectrometry (MS) analysis.

Strep-Flag tagged CENP-A or CG2051, respectively, were purified by incubating nuclear extracts with Strep-Tactin Superflow resin (50% slurry in Buffer N; IBA) at ~150 µl slurry per 1 ml extract for 2 h with rotation. Beads were collected by centrifugation at 700xg for 3 min and washed several times with Buffer N. Proteins were eluted twice with 200 µl TBS buffer (30 mM Tris/HCl, pH 7.4, 150 mM NaCl, 1 mM EDTA) containing 2.5 mM desthiobiotin. Subsequently, eluates were incubated with 100 µl FLAG-M2-agarose (50% slurry in TBS buffer; Sigma) for 2 h with rotation. Beads were collected by centrifugation as above, washed sequentially with TBS/0.1% NP-40, TBS and a.dest. and eventually eluted with 5% acetic acid and 5% acetic acid/10% acetonitrile. For the analysis of posttranslational modifications, CENP-A was purified in an identical manner, except that the starting material was either nuclear extract or cytoplasmic extract and that the final eluates were separated on an SDS-PAGE gel.

Purification from bacteria: Expression of recombinant proteins was induced with 1 mM IPTG for 2 h at 37 °C. Cells were harvested, and lysed by sonication in lysis buffer (10 mM Tris/HCl pH 8, 200 mM NaCl, 0.05% Tween 20, 0.1 mM PMSF, 10 mM imidazole). After centrifugation at 14,000 rpm for 20 min at 4°C, extract containing His-CENP-A was either used directly for Ni^{2+}-NTA agarose purification or was combined with bacterial extract containing SF-CG2051 (1:1) and incubated at 25°C for 1 h with gentle agitation followed by incubation with pre-equilibrated Ni^{2+}-NTA agarose beads (Qiagen) for 4 h at 4°C. After extensive washing with 20 mM imidazole in lysis buffer, beads were eluted with elution buffer (lysis buffer containing 50 mM NaCl and 300 mM imidazole). One

column volume fractions were collected. To control for unspecific binding, SF-CG2051-containing extract was mixed with lysis buffer instead of His-CENP-A extract and purified in the same way.

Quantification of immunoblot signals

To determine the relative distribution of CG2051/Hat1-CENP-A complexes, signal intensities of Hat1 and CENP-A bands were determined after immunoblotting using ImageJ. Taking into account gel loading volume and total extract volume, the absolute amount of protein in a particular cell fraction was calculated and the relative distribution was determined. To calculate the proportion of Hat1 that is associated with CENP-A in each cellular fraction, total amount of protein (arbitrary units) was calculated from signal intensity of the input lane and total volume of input. The signal intensity in the beads lane was then used to calculate the proportion of total protein that was precipitated with CENP-A.

Mass spectrometry and data processing

To identify interaction partners of CENP-A or CG2051/Hat1, the respective proteins were purified by affinity chromatography and elutions were concentrated by speed vac evaporation. Samples were redissolved in 65 µl 100 mM NH_4HCO_3/3.8 mM DTT, pH 8.0 and incubated at 56°C for 30 min. 25 µl of 55 mM iodoacetamide (IAA)/100 mM NH_4HCO_3 was added and incubated at room temperature in the dark before the sample was digested with 1 µg trypsin (Sigma Aldrich) at 37°C for 6 hours. Resulting peptides were separated on a homemade fritless fused-silica microcapillary column (75 µm i.d. x 280 µm o.d. x 10 cm length) packed with 5 µm reversed-phase C18 material (Reprosil). Solvents for HPLC were 0.1% formic acid (solvent A) and 0.1% formic acid in 85% acetonitrile (solvent B). The gradient profile was as follows: 0-2 min, 4% B; 2-117 min, 4-50% B; 117-122 min, 50-100% B, and 122-27 min, 100% B. The flow rate was 300 nl/min. Samples were analysed using an UltiMate 3000 nano-HPLC system (Thermo Scientific) coupled to a LTQ Orbitrap XL mass spectrometer (Thermo Scientific). The instrument was operated in the data dependent mode selecting the top 10 most abundant isotope patterns with charge 2+ and 3+ from the survey scan with an isolation window of 2 mass-to-charge ratio (m/z). Survey full scan MS spectra were acquired form 300 to 1800 m/z at a

resolution of 60,000 with a maximum injection time (IT) of 20 ms, and automatic gain control (AGC) target 1e6. The selected isotope patterns were fragmented by collisional induced dissociation (CID) with normalized collision energy of 35 and a maximum injection time of 55 ms. Data analysis was performed using Proteome Discoverer 1.3 (Thermo Scientific) with search engine Sequest. The raw files were searched against the *Drosophila melanogaster* BDGP5.68 pep.all database (21,076 entries). Precursor and fragment mass tolerance was set to 10 ppm and 0.8 Da, respectively, and up to two missed cleavages were allowed. Carbamidomethylation of cysteine and oxidation on methionine were set as variable modifictions. Peptide identifications were filtered at 1% false discovery rate.

For analysis of posttranslational modifications, CENP-A protein bands were excised from an SDS-gel, digested with trypsin or LysC as previously described (Sobieszek et al. 2006) and analyzed using an UltiMate 3000 nano-HPLC system (Thermo Scientific) coupled to a Q Exactive Plus mass spectrometer (Thermo Scientific) equipped with a Nanospray Flex ionization source. The peptides were separated on a homemade fritless fused-silica microcapillary column (75 µm i.d. x 280 µm o.d. x 10 cm length) packed with 3 µm reversed-phase C18 material (Reprosil). Solvents for HPLC were 0.1% formic acid (solvent A) and 0.1% formic acid in 85% acetonitrile (solvent B). The gradient profile was as follows: 0-2 min, 4% B; 2-55 min, 4-50% B; 55-60 min, 50-100% B, and 60-65 min, 100 % B. The flow rate was 250 nl/min. The Q Exactive Plus mass spectrometer was operated in the data dependent mode selecting the top 12 most abundant isotope patterns with charge >1 from the survey scan with an isolation window of 1.6 mass-to-charge ratio (m/z). Survey full scan MS spectra were acquired from 300 to 1750 m/z at a resolution of 70,000 with a maximum injection time (IT) of 120 ms, and automatic gain control (AGC) target 1e6. The selected isotope patterns were fragmented by higher-energy collisional dissociation (HCD) with normalized collision energy of 25 at a resolution of 17,000 with a maximum IT of 120 ms, and AGC target 5e5. Data analysis was performed using Proteome Discoverer 1.4.1.14 (Thermo Scientific) with search engine Sequest. The raw files were searched against the *Drosophila melanogaster* BDGP5.68 pep.all database. Precursor and fragment mass tolerance was set to 10 ppm and 0.02 Da, respectively, and up to two missed cleavages were allowed. Carbamidomethylation of cysteine was set as static modification, oxidation of methionine,

phosphorylation of serine, threonine and tyrosine, methylation and dimethylation of lysine and arginine, trimethylation of arginine and acetylation of lysine were set as variable modifications. Peptide identifications were filtered at 1% false discovery rate.

Quench-chase-pulse labeling of newly synthesized CENP-A

To quench SNAP tag activity of existing SNAP-CENP-A, old growth medium was removed, and S2 cells were incubated with 0.9 µM SNAP-Cell Block (NEB) in fresh S2 medium for 30 min at 25°C. Cells were then washed four times (10 min each) in fresh medium before addition of conditioned S2 medium supplemented with 5 µM (Figure 7, Supplementary Figure S6) or 10 µM (Supplementary Figure S6) $CuSO_4$ to induce production of SNAP-CENP-A. After 24 h (Supplementary Figure S6) or 48 h (Figure 7, Supplementary Figure S6) chase period. Cells were transferred to concanavalin-A coated coverslips in 24-well plates and left to settle for 20 min. Medium was removed and pulse-labelling of newly synthesized SNAP-CENP-A was performed by incubation with 4.5 µM SNAP-Cell TMR Star (NEB) in fresh medium for 30 min. Non-reacted TMR Star was removed by washing cells 3 times for 10 min each with fresh medium before fixation in 5% paraformaldehyde/0.3% Triton-X100 in PBS. After additional washing in PBS, nuclei were counterstained with DAPI and cells were mounted in vectashield for confocal fluorescence microscopy on a Leica TCS SP5 instrument. Image aquisition was performed using a 63x oil objective with a pixel size of 48.1 nm and by collecting 0.13 µm z-sections spanning the entire nuclei. 3D images were reconstructed and analysed by Imaris v5.1. Using the new Arena function of Imaris we have generated an "assay" grouping all images into either control or Hat1 group. One image of the Hat1 group was selected and spots were created on that image. The obtained parameters (including intensity and quality thresholds) for Spot creation were saved ("save for batch") and thereafter applied in the same way to both control and Hat1 images to ensure absolutely consistent image processing. The analyses were performed with and without prior deconvolution using Huygens software. Since the results were virtually the same, only the data without deconvolution are shown. A total of 235-638 centromeres per experiment and treatment condition was analysed in this manner. For comparison of Hat1 RNAi and control samples, mean

TMR ± SEM intensities were calculated and statistical significance was determined by two-tailed t-test and Mann-Whitney-test using Prism5.0 software.

Supplementary Figures

```
CLUSTAL 2.1 multiple sequence alignment

Human Hat1              ---MAGFGAMEKFLVEY----KSAVEKKLAEYKCNTNTAIELKLVRFPEDLENDIRTFFP 53
M. musculus Hat1        ------MAALEKFLVEY----KSAVEKKLAEYKCNTNTAIELKLVRFPEDLENDIRTFFP 50
G. gallus Hat1          --------------MAG----LTPMEKKLAEYKCNTNEAIQLKLVRFPEDLEDDNTTFNP 42
X. tropicalis Hat1      MANEEYKAQTERTRLPDSLLGLSAMEKKLAEYKCDTNEAIKLKLVRFPEDIEDESMTFNP 60
D. rerio Hat1           --------------MAA----VNDMEKKLAEYKCDTNEAICLKLVRFPEDIDDESTTFHP 42
D. melanogaster CG2051  ----------------------MAQIEKYQDFVIDALEVVDFKLIRDKADINNDALTFHP 38
S. cerevisiae Hat1      ---------------------MSANDFKPETWTSSANEALRVSIVGEN------AVQFSP 33
S. pombe Hat1           ---------------------MSA----VDEWVHNANECIEIVQVNEKHEK---DCQYHP 32

Human Hat1              EYTHQLFGDDETAFGYKGLKILLYYIAGSLSTMFRVEYASKVDENFD-CVEADDVEGKIR 112
M. musculus Hat1        EYTHQLFGDDETAFGYKGLKILLYYIAGSLSTLFRVEYSSKVDENFD-CVEADDVEGKIR 109
G. gallus Hat1          EYSHQVFGDDEVAFGYKGLKILLYYIAGNLSTLFRIEYTSKVNEKFD-CVEADDVESKIR 101
X. tropicalis Hat1      EYSHQIFGDEEVAFGYKGLKILLYYSAGNLSNMFRVEYSSKVDDKFD-LVQPDDVENKIR 119
D. rerio Hat1           EFSHQLFGDDEVAFGYKGLQIQLYQSAGNLNTLFKVKYTARVTDKFD-CVEPDDVEGKIR 101
D. melanogaster CG2051  AMAHQIFGETETIFGYQDLHVRVMYTAGPLHIYLGVDYGKRVNEISGGEIKADDVVSTIA 98
S. cerevisiae Hat1      LFTYPIYGDSEKIYGYKDLIIHLAFDSVTFKPYVNVKYSAKLGDDN-----IVDVEKKLL 88
S. pombe Hat1           SNTYAIFGDAEVIYGYKDLNVTITYECPLMVPKLEISYSERLAPDSG--VEPTDIEGTLN 90

Human Hat1              QIIPPG-FCTNTNDFLSLLEKEVDFKPFGTLLHTYSVLSPTGGENFTFQIYKADMTCRGF 171
M. musculus Hat1        QIIPPG-FCTNTNDFLSLLEKETNFKPFGTLLHTYTVPSQTGGETFTFQIHKADMTCRGF 168
G. gallus Hat1          EIIPPG-FCSNTDDFVSLLEKEVNFKPFGMLLHTYSVHNEEAGEDITYQIYKADMTCPGF 160
X. tropicalis Hat1      ELIPSG-FCCGSDDFISLLEKEVNFKPMGTVVHTYTVHNEEAGEDITYQIYKADMTCPGF 178
D. rerio Hat1           EIIPAG-FCCNTDDFISVLEKEANFKPFGSLQHTYKVHNVEAGEDLTYQIHKVDISCPGF 160
D. melanogaster CG2051  QSLPDGCYFINLDEFLKTLDKADKFQPFGEKISEYRRVSDDGSE-RLFEIYQCEYKSSSF 157
S. cerevisiae Hat1      SFLPKD--DVIVRDEAKWVDCFAEERKTHNLSDVFEKVSEYSLNGEEFVVYKSSLVDDFA 146
S. pombe Hat1           TYLK----DRSIKVEGNSFDVHSAN-----------SIHNYSFNGKTFKILQATVLE--A 133

Human Hat1              REYHERLQTFLMWFIETASFIDVDDERWHYFLVFEKY-NKDGATLFATVGYMTVYNYYVY 230
M. musculus Hat1        REYHERLQTFLMWFIETASFIDVDDERWHYFLVFEKY-NKDGATLFATVGYMTVYNYYVY 227
G. gallus Hat1          REYHERLQTFLMWFIETASFIDVDDERWNYFIVFEKY-NKDGATLFATVGYMTVYNYYVY 219
X. tropicalis Hat1      REYHERLQTFLMWFIETASYIDVDDEKWDYFLVFEKY-NKDGATLYATVGYMTVYNYYVY 237
D. rerio Hat1           REYHERLQTFLMWFIETASFIDVDDDRWDFFLVFEKY-NKDGETLYATVGYMTVYNYYVY 219
D. melanogaster CG2051  LKFFARLQTFILWFVDAASYIDTDDPQWCYFLSYEKYKNNDGQWQYATAGYTTVYEYYAY 217
S. cerevisiae Hat1      RRMHRRVQIFSLLFIEAANYIDETDPSWQIYWLLN-------KKTKELIGFVTTYKYWHY 199
S. pombe Hat1           SEIMQHLQIFSLFFIEGGSFIDLNDPRWMVYLLYETT-----EDDYCLRGYCTVYKYYK- 187

Human Hat1              P----------DKTRPRVSQMLILTPFQGQGHGAQLLETVHRYYTEFPTVLDITAEDPSK 280
M. musculus Hat1        P----------DKTRPRVSQMLILTPFQGQGHGAQLLETVHRYYISFPTVLDITAEDPSR 277
G. gallus Hat1          P----------DKTRPRVSQMLILPPFQGEGHGAQLLETVHRYYMSSPTVLDITAEDPSE 269
X. tropicalis Hat1      P----------DKTRPRVSQMLVLPPFQGEGHGAQLLETVHRYYVSASNILDITAEDPSE 287
D. rerio Hat1           P----------DKTRPRVSQMLILPPFQGEGHGAQLLETVHRYYCTSPKVQDITAEDPSE 269
D. melanogaster CG2051  P----------QNKRPRISQMLILPPFQKLGLATQLVESIYRFYQSQKNVVDITVEDPSE 267
S. cerevisiae Hat1      LGAKSFDEDIDKKFRAKISQFLIFPPYQNKGHGSCLYEAIIQSWLEDKSITEITVEDPNE 259
S. pombe Hat1           -----WDKLIHDGIRARISQFVILPPFQHQGHGSQLYNAIVSTFLKNPKILDFTVEDASE 242

Human Hat1              SYVKLRDFVLVKLCQDLPCFSREKLMQGFNEDMAIEAQQKFKINKQHARRVYEILRLLVT 340
M. musculus Hat1        SYLKLRDFVLVKFCQFLPSFSRERLLQGFSEDMAIQAQQMFKINKQHARRVYEILRLLVT 337
G. gallus Hat1          NYIKLRDFVLVKLCQDLPCFSPAKLMQGFSQEMVMEAQQKLKINKQHTRRVYEILRLRAT 329
X. tropicalis Hat1      NFVRLRDFVLVKLCQNLPCFSLEKLLCGFSQEMVTEAQEKLKINKKHARRVYEILRLRAT 347
D. rerio Hat1           NYVKLRDFVLVKLCLTLPSFASEKLSQGFSEEMASEAREKLKINKKHARRVYEILRLRNT 329
D. melanogaster CG2051  DFQRLRNFVDARSCMKLKSFAPGEIVKGFNKEMVREAREALKLNPLQVRKVYELLRLFYT 327
S. cerevisiae Hat1      AFDDLRDRNDIQRLRKLGYDAVFQKHSDLSDEFLESSRKSLKLEERQFNRLVEMLLLLNN 319
S. pombe Hat1           AFDSLRDHCDYKRLLSMGIFSEPDFHPSLSRQWINSKIAETKLTQRQFSRCCELAFTTKL 302

Human Hat1              DMSDAEQYRSYRLDIKRRLISPYKKKQRDLAKMRKCLRPEELTNQMNQIEISMQHEQLEE 400
M. musculus Hat1        DMSDAEQYRSYRLDIKRRLISPYKKKQRDLAKMKKCLRPEELTNQMNQIEISVQHEQLEE 397
G. gallus Hat1          DMGDAEQSRSYRLDVKRRLIGPYKKKQRELAKMRRCLRPEELTNQLNQIDLNMQHEQLEE 389
X. tropicalis Hat1      DMSDSEKAKAYRLDVKKRLVGPYKKNQREMAKMKQCLRPEELTNQMNQIDLNTQHEQLEE 407
D. rerio Hat1           DMNDEKKAREFRLEVKKRLFGPYRKNQRELTKMRKCLRPEELASHISQMDTTLQHEELEK 389
D. melanogaster CG2051  NVKDEKEYRPYRLEVKKRLNAVYYKQLKDLKKMERFKMDTEMLRAR-LPTVQQRMEQLHE 386
S. cerevisiae Hat1      S-------PSFELKVKNR---LYIKNYDALDQTDPEKAREALQNSF--ILVKDDYRRIIE 367
S. pombe Hat1           KKLSLLERKSVRLGIKER---IFRQNLDVLLQLDKSERIEKIHNAY--ENQFDEYKQIVK 357

Human Hat1              SFQELVEDYRRVIERLAQE-- 419
M. musculus Hat1        RFQELVEDYRRVIERLAQE-- 416
G. gallus Hat1          SFQQLVSDYRRVLERLAQA-- 408
X. tropicalis Hat1      SYQQLVEEYRRIVERLAQA-- 426
D. rerio Hat1           SYQDVVAEYRRVLERLAQV-- 408
D. melanogaster CG2051  EYRVVEQEYQSTIAKLRAS-- 405
S. cerevisiae Hat1      SINKSQG-------------- 374
S. pombe Hat1           KLPKLKEDSPRKRQKLAQSSS 378
```

Supplementary Figure S1. Multiple sequence alignment of *Drosophila* CG2051 with Hat1 proteins from other species. Amino acids conserved in at least two species including *Drosophila* are highlighted in red; amino acids conserved in at least two species but not in *Drosophila* are denoted in blue and green, respectively.

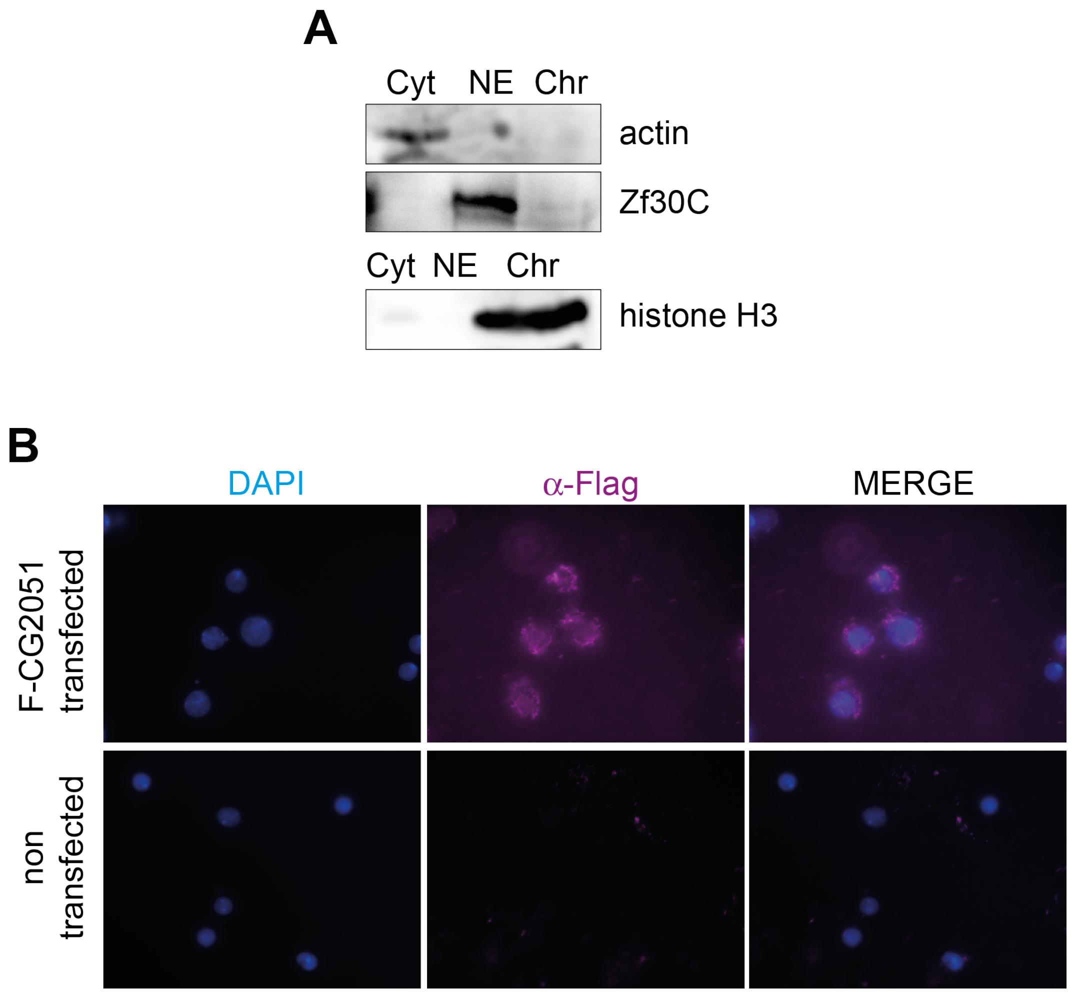

Supplementary Figure S2. (A) Control western blot to determine the quality of biochemical cell fractionation. Cytoplasmic (Cyt), nuclear (NE) and chromatin (Chr) extracts (15 µg / 8 µg / 11 µg total protein) were western blotted and incubated with antibodies against the following marker proteins: actin (cytoplasm), Zinc-finger transcription factor Zf30C (nuclear extract) and histone H3 (chromatin protein). (B) Immunostaining of S2 cells expressing Flag-tagged CG2051 (upper panels) and non-transfected S2 cells (lower panels) with α-Flag antibodies (magenta) to demonstrate the specificity of the Flag antibody. DNA was visualized by DAPI staining (blue).

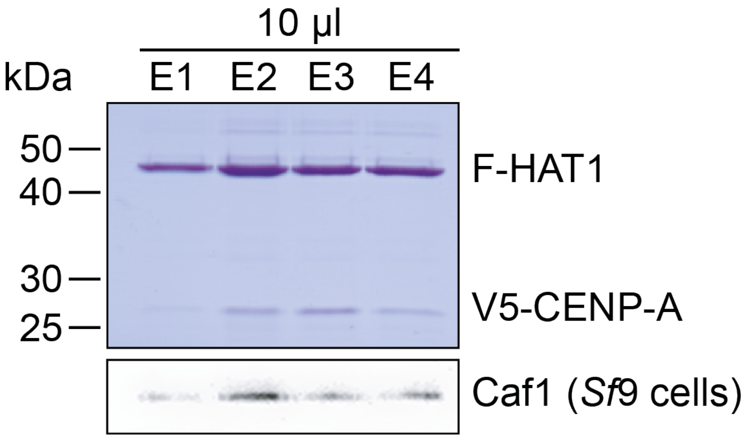

Supplementary Figure S3. Flag-tagged *Drosophila* Hat1 and V5-tagged *Drosophila* CENP-A were overexpressed in *Sf*9 cells using the baculovirus system and subsequently purified by Flag-affinity chromatography. Aliquots of elutions E1-E4 were subjected to SDS PAGE and Coommassie staining or western blot with anti-Caf1 antibodies. Thereby, co-purification of endogenous (i.e. *Sf*9 cell) Caf1 with Hat1-CENP-A complexes was detected.

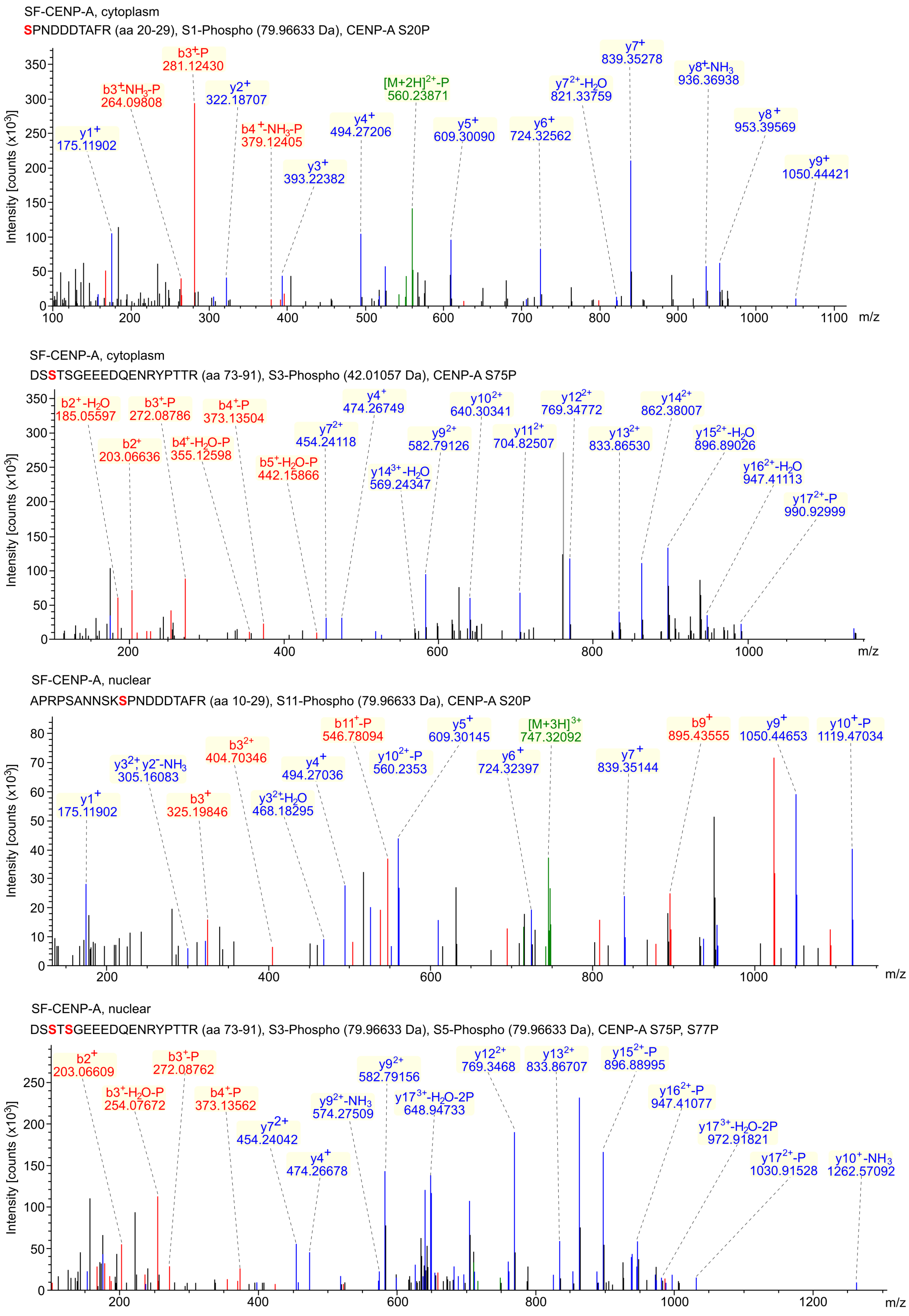

Supplementary Figure S4. MS spectra of peptides obtained by trypsin or LysC digestion of cytoplasmic (*upper* two panels) and nucleoplasmic (*lower* two panels) CENP-A showing phosphorylation on serine residues. For each modified residue one example spectrum is shown, although usually several spectra were obtained due to differential phosphorylation (e.g. mono-, di-

phosphorylation of S75/S77) or missed cleavage by the proteases. Peptide sequences are shown on top of each panel. The positions of the respective amino acids in full length CENP-A are noted in parentheses.

A **Peptide sequence**

H4 peptide	N-SGRGKGGKGLGKGGAKRH-C
CENP-A (N)	N-PRHSRAKRAPRPSANNS-C
CENP-A (M)	N-RMTVQQESKTRAAGPVA-C

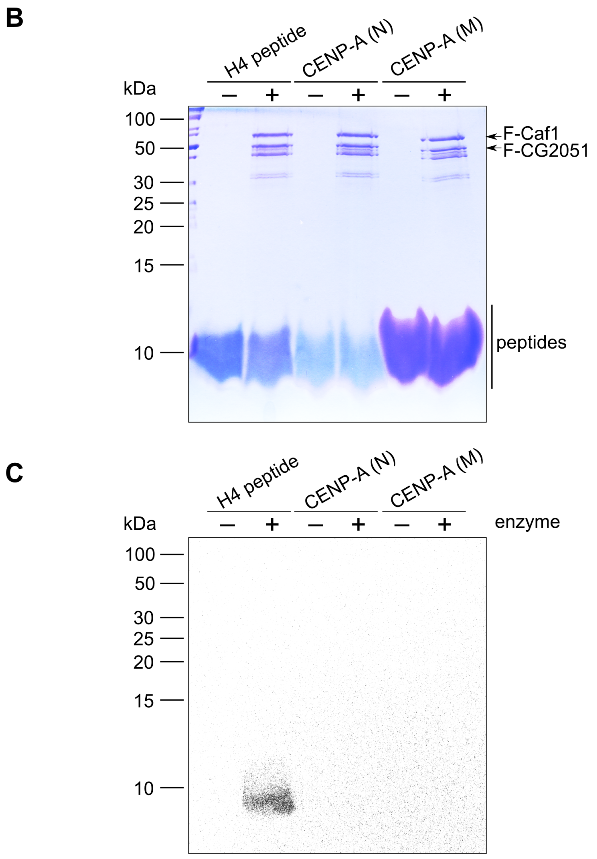

Supplementary Figure S5. CG2051 does not acetylate CENP-A-derived peptides. (A) Sequences of peptides used in HAT assays. (B) Recombinant purified CG2051 and Caf1 were incubated with the different peptides in the presence of ^{14}C-acetyl-CoA and reactions were subsequently separated by 18% SDS-PAGE. The gel was stained with Coommassie brilliant blue, dried and exposed to phosphoimage screen. (C) Acetylation signal on the H4 peptide, but not on any of the CENP-A peptides detected after phosphoimaging of the gel shown in (B).

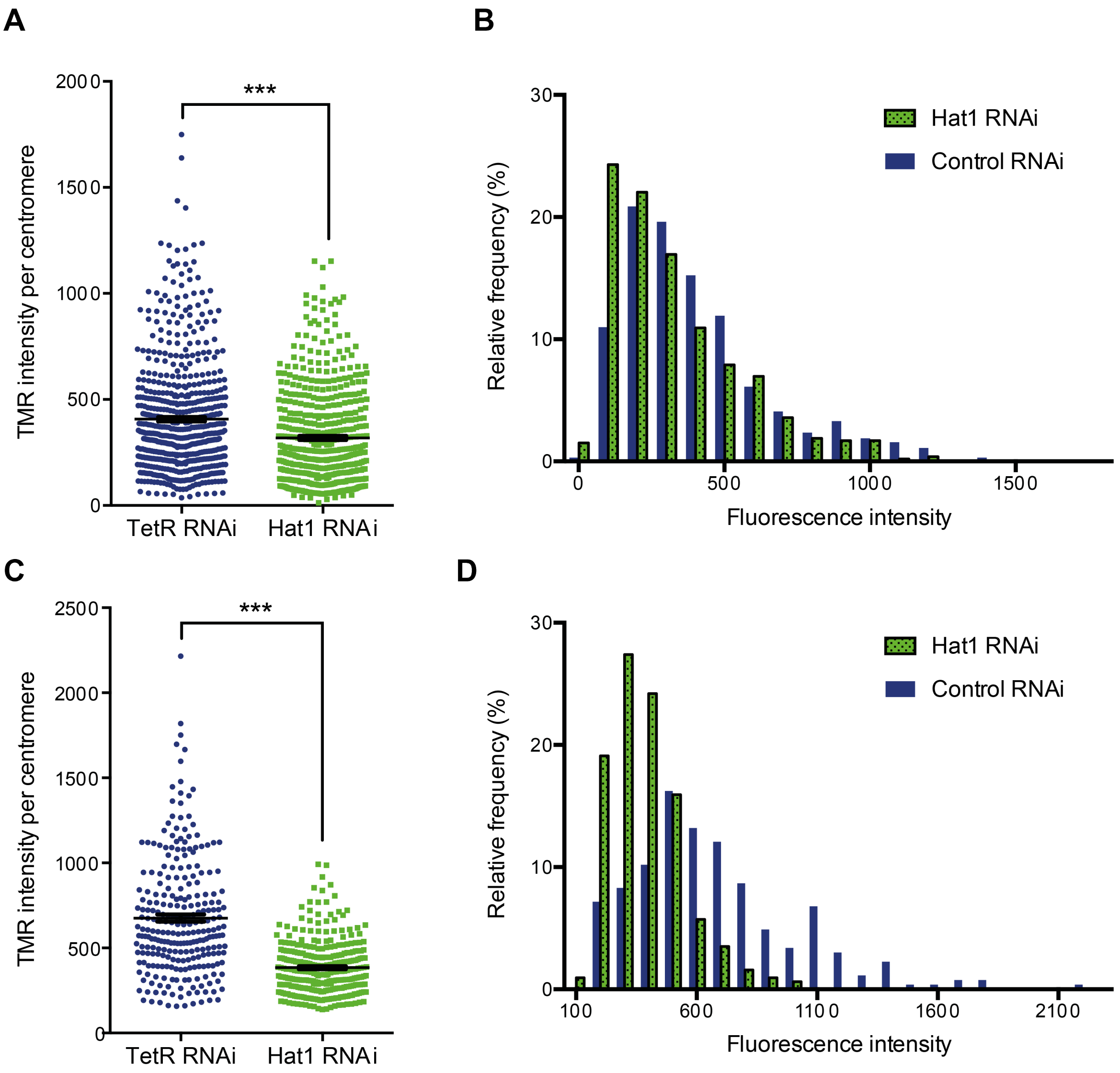

Supplementary Figure S6. CENP-A incorporation defect after Hat1 RNAi treatment of S2 cells. (A, C) Quantification of SNAP-CENP-A intensities in Hat1 knock-down and TetR RNAi control cells of two independent experiments using Imaris v5.1 software. Statistical significance was determined by unpaired t-test and Mann-Whitney-test (*** $p< 0.0001$). In (A, B) dsRNA treatment was for 4 days before SNAP signal blocking and induction of CENP-A with 10 µM $CuSO_4$ for 24 h; in (C, D) cells underwent RNAi for 6 days before CENP-A was induced with 5 µM $CuSO_4$ for 48 h. (B, D) Histograms showing the distribution in intensity of centromeric SNAP-CENP-A signals in Hat1 RNAi and control cells in the experiments quantified in (A and C).

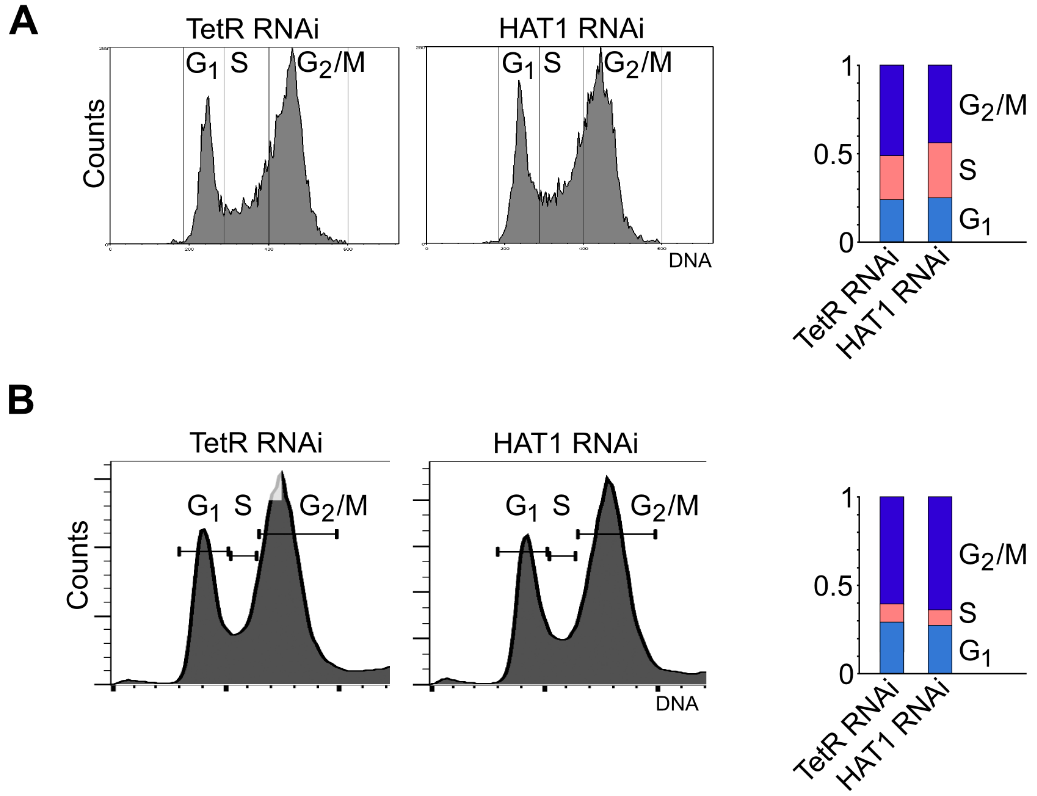

Supplementary Figure S7. Cell cycle profile analysis of Hat1 RNAi-treated S2 cells. FACS analysis of propidium iodide stained cells was performed on cells treated with dsRNA against TetR (control) or Hat1 for 4 days. FACS profiles are shown in the left two panels and the percent distribution of cell cycle phases is shown in the diagram at the right.
